# Circulating Tumor DNA and Survival in Metastatic Breast Cancer: A Systematic Review and Meta-Analysis

**DOI:** 10.1001/jamanetworkopen.2024.31722

**Published:** 2024-09-05

**Authors:** Kyle Dickinson, Archi Sharma, Ramana-Kumar Venkata Agnihotram, Selin Altuntur, Morag Park, Sarkis Meterissian, Julia V. Burnier

**Affiliations:** 1 Cancer Research Program, Research Institute of the McGill University Health Centre, Montreal, Quebec, Canada; 2 Biostatistics Consulting Unit, Research Institute of the McGill University Health Centre, Montreal, Quebec, Canada; 3 McConnell Resource Centre Medical Library, McGill University Health Centre, Montreal, Quebec, Canada; 4 Rosalind and Morris Goodman Cancer Institute, McGill University, Montreal, Quebec, Canada; 5 Gerald Bronfman Department of Oncology, McGill University, Montreal, Quebec, Canada; 6 Department of Surgery, McGill University Health Centre, Montreal, Quebec, Canada; 7 Department of Pathology, McGill University, Montreal, Quebec, Canada

## Abstract

**Importance:**

Metastatic breast cancer (MBC) poses a substantial clinical challenge despite advancements in diagnosis and treatment. While tissue biopsies offer a static snapshot of disease, liquid biopsy—through detection of circulating tumor DNA (ctDNA)—provides minimally invasive, real-time insight into tumor biology.

**Objective:**

To determine the association between ctDNA and survival outcomes in patients with MBC.

**Data Sources:**

An electronic search was performed in 5 databases (CINAHL, Cochrane Library, Embase, Medline, and Web of Science) and included all articles published from inception until October 23, 2023.

**Study Selection:**

To be included in the meta-analysis, studies had to (1) include women diagnosed with MBC; (2) report baseline plasma ctDNA data; and (3) report overall survival, progression-free survival, or disease-free survival with associated hazards ratios.

**Data Extraction and Synthesis:**

Titles and abstracts were screened independently by 2 authors. Data were pooled using a random-effects model. This study adhered to the Preferred Reporting Items for Systematic Reviews and Meta-analyses (PRISMA) reporting guideline, and quality was assessed using the Newcastle-Ottawa Scale.

**Main Outcomes and Measures:**

The primary study outcome was the association between detection of specific genomic alterations in ctDNA with survival outcomes. Secondary objectives were associations of study methodology with survival.

**Results:**

Of 3162 articles reviewed, 37 met the inclusion criteria and reported data from 4264 female patients aged 20 to 94 years. Aggregated analysis revealed a significant association between ctDNA detection and worse survival (hazard ratio, 1.40; 95% CI, 1.22-1.58). Subgroup analysis identified significant associations of *TP53* and *ESR1* alterations with worse survival (hazard ratios, 1.58 [95% CI, 1.34-1.81] and 1.28 [95% CI, 0.96-1.60], respectively), while *PIK3CA* alterations were not associated with survival outcomes. Stratifying by detection method, ctDNA detection through next-generation sequencing and digital polymerase chain reaction was associated with worse survival (hazard ratios, 1.48 [95% CI, 1.22-1.74] and 1.28 [95% CI, 1.05-1.50], respectively).

**Conclusions and Relevance:**

In this systematic review and meta-analysis, detection of specific genomic alterations in ctDNA was associated with worse overall, progression-free, and disease-free survival, suggesting its potential as a prognostic biomarker in MBC. These results may help guide the design of future studies to determine the actionability of ctDNA findings.

## Key Points

**Question:**

What is the association between detection of specific alterations in circulating tumor DNA (ctDNA) and survival in patients with metastatic breast cancer?

**Findings:**

In this systematic review and meta-analysis reporting data from 4264 patients in 37 studies, detection of ctDNA was associated with worse overall, progression-free, and disease-free survival in patients with metastatic breast cancer. This association remained significant irrespective of differences in study design, including ctDNA detection method and type of blood collection tubes.

**Meaning:**

These findings suggest the potential of ctDNA detection as a prognostic biomarker in patients with metastatic breast cancer and may help guide the design of future clinical studies.

## Teaser

This systematic review and meta-analysis examines the association between circulating tumor DNA detection and survival outcomes in patients with metastatic breast cancer.

## Introduction

Metastatic breast cancer (MBC) has a very poor prognosis, with an average survival of 2 to 3 years, posing a significant clinical challenge due to low response rates to existing therapies.^1^ Current diagnostic and surveillance methods for MBC rely heavily on imaging modalities and function tests, which often detect lesions that developed months or years before the test. Tissue biopsies of metastatic lesions offer insights into the molecular profile of the tumor; however, these biopsies are invasive, introducing risks and morbidity, and cannot be performed longitudinally to monitor disease changes, treatment response, and progression.^2^ To overcome this challenge, liquid biopsy has emerged as a valuable tool for diagnosing and monitoring treatment response in various cancers. The minimally invasive nature of liquid biopsy allows for more frequent sample collection, offering a comprehensive view of an individual’s disease compared with a single tissue biopsy. A key analyte in liquid biopsy is circulating tumor DNA (ctDNA), representing small fragments of cell-free DNA (cfDNA) released mainly by apoptotic or necrotic tumor cells into the circulation.^3^ Due to its relatively short half-life in the blood, ctDNA provides real-time information on treatment response, tumor biology, and evolution.

*TP53* and *PIK3CA* emerge as the most frequently reported driver alterations in primary breast cancer; however, in MBC, additional alterations in different metastatic sites add complexity to the overall genetic profile.^4^ Monitoring techniques currently used in practice, though, lack the capability to identify the onset of disease progression and treatment resistance at an early stage, thus hindering the timely transition to a more potent and effective treatment strategy. Therefore, using ctDNA to track the variant profile of a tumor in real time holds considerable implications for the clinical management of MBC.

While promising data on ctDNA in MBC exist,^5,6,7,8,9,10,11,12,13,14,15,16,17,18,19,20,21,22,23,24,25,26,27,28,29,30,31,32,33,34,35,36,37,38,39,40,41^ inconsistencies among studies regarding study design, cohort size, analysis methods, and other factors have led to conflicting conclusions. Consequently, a systematic review of the current literature is needed to establish the association of ctDNA with clinical parameters and to identify the methodological factors influencing the findings. As such a review has not yet been conducted in the MBC setting, we investigated the association between detection of specific alterations in ctDNA and overall survival (OS), progression-free survival (PFS), and disease-free survival (DFS) in patients with MBC. In secondary analyses, we evaluated the association of various elements of clinical study design with the correlation between ctDNA and survival outcomes. By evaluating the influence of detecting specific ctDNA alterations and considering differences in study design, we may better understand the prognostic ability of ctDNA in MBC.

## Methods

### Search Strategy and Study Selection

This systematic review and meta-analysis adhered to the Preferred Reporting Items for Systematic Reviews and Meta-Analyses (PRISMA) reporting guideline. Patient consent and ethnical approval were not required for this study as the data and results were extracted from previously approved studies.

An electronic search of the following databases was performed: CINAHL, Cochrane Library, Embase, Medline, and Web of Science. The search strategies used in each database were formulated with help from the McGill University Health Centre Library and are described in detail in eTable 1 in Supplement 2. All studies were screened from database inception to October 23, 2023. Articles were imported into EndNote, version 20 (Clarivate), and duplicates were removed. The full texts of selected articles were downloaded and reviewed to determine eligibility. To be included in the analysis, we selected clinical studies (prospective or retrospective) that (1) included women (aged ≥18 years) diagnosed with MBC, advanced breast cancer, or stage IV breast cancer; (2) reported baseline plasma ctDNA data; and (3) reported OS, PFS, or DFS with associated hazards ratios. Non–English-language studies, conference abstracts, review articles, studies with no sample size given for the survival analysis, case reports or series, ctDNA results from body fluids other than plasma, and studies that did not report survival outcomes using hazard ratios were excluded. Titles and abstracts were independently screened by K.D. and A.S. The review was not registered.

### Data Extraction and Quality Assessment

The following variables were extracted from the included articles: title, sample size, survival outcome (hazard ratio including 95% CI and *P* value), study design (prospective or retrospective), breast cancer subtype, timing of blood draw (before or after treatment), target alteration, method of ctDNA analysis, blood tube used, cfDNA extraction method (column or magnetic bead), and primary objective of the study (ie, whether ctDNA was analyzed as a primary or secondary objective). Data tables used for the analysis are available upon request.

The Newcastle Ottawa Scale was used for the quality assessment of the included studies. Newcastle Ottawa Scale scores for all included studies and a description of the scoring system are provided in eTable 2 in Supplement 2.

### Statistical Analysis

Forest plots were used to visually represent pooled hazard ratios alongside their corresponding 95% CIs using a random-effects model. To gauge statistical heterogeneity, the inconsistency index (*I*^2^) was used. Examination for publication bias was conducted through a visual assessment using a funnel plot. All statistical analyses, including sensitivity analyses, were performed using Stata, version 14.2 (StataCorp LLC).

## Results

### Eligible Studies

**Figure 1.**
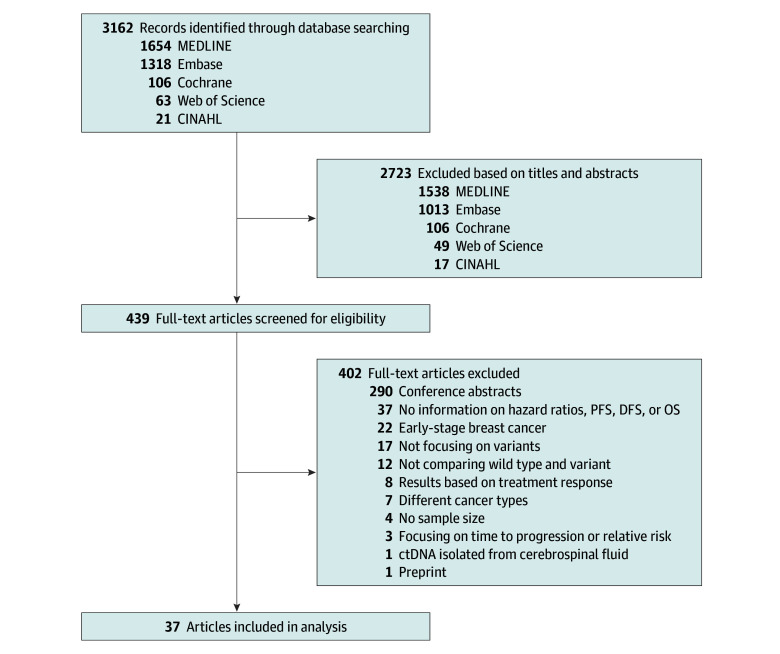
PRISMA Flow Diagram of the Literature Search and Study Selection ctDNA indicates circulating tumor DNA; DFS, disease-free survival; OS, overall survival; PFS, progression-free survival.

**Figure 2.**
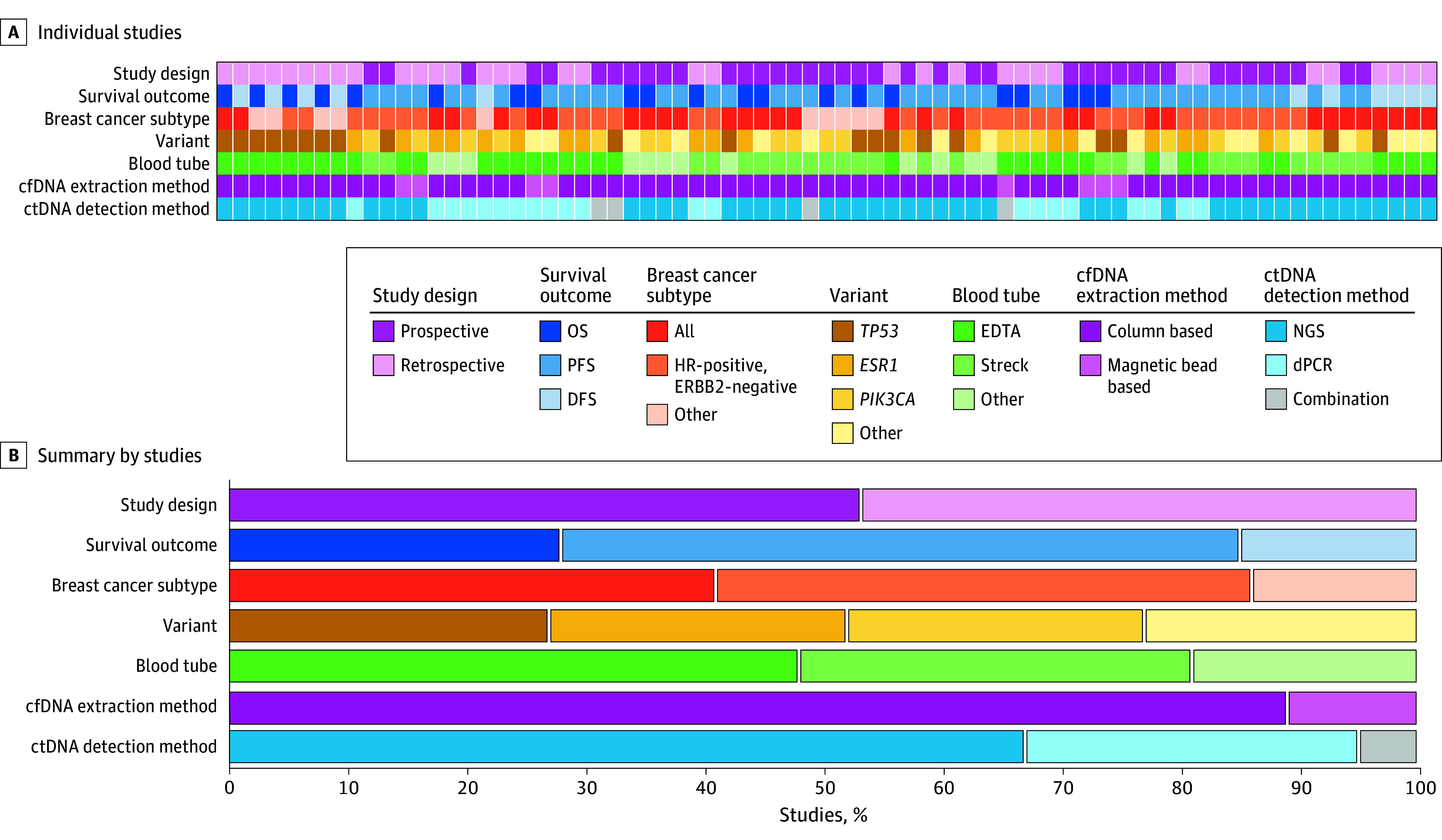
Visual Summary of Study Characteristics cfDNA indicates cell-free DNA; ctDNA, circulating tumor DNA; DFS, disease-free survival; dPCR, digital polymerase chain reaction; HR, hormone receptor; NGS, next-generation sequencing; OS, overall survival; PFS, progression-free survival.

A total of 3162 publications were identified using our comprehensive search string (eTable 1 in Supplement 2). After applying our inclusion and exclusion criteria, we included 37 publications (1.2%) reporting data from 4264 female patients aged 20 to 94 years with MBC or stage IV breast cancer (Figure 1).^5,6,7,8,9,10,11,12,13,14,15,16,17,18,19,20,21,22,23,24,25,26,27,28,29,30,31,32,33,34,35,36,37,38,39,40,41^ Of the 37 included studies, 20 (54%) were prospective^7,10,14,15,16,18,19,20,21,23,28,29,30,31,32,33,35,36,37,38^ and 17 (46%) were retrospective.^5,6,8,9,11,12,13,17,22,24,25,26,27,34,39,40,41^ Some studies featured multiple survival analyses,^5,7,8,9,13,14,15,16,17,19,21,23,24,26,30,34,35,36,38,41^ each distinct in terms of survival outcomes, detected variants, or breast cancer subtype. Consequently, each of these analyses were treated as an individual study for a total of 75 studies in our meta-analysis extracted from the 37 articles. Study and patient characteristics are reported in Table 1 and represented visually in Figure 2.

**Table 1.** Summary of Study and Patient Characteristics^a^

Source	Target alteration	Breast cancer subtype	Outcome tested	Sample size	Before or after treatment	Method of ctDNA analysis	Study design	Blood tube used
Bai et al,^5^2021	*TP53*	All	OS	195	Before	NGS	Retrospective	EDTA
	*TP53*	All	DFS	148	Before	NGS	Retrospective	EDTA
	*TP53*	ERBB2 (formerly HER2)-positive	OS	37	Before	NGS	Retrospective	EDTA
	*TP53*	HR-positive, ERBB2-negative	OS	113	Before	NGS	Retrospective	EDTA
	*TP53*	HR-positive, ERBB2-negative	DFS	89	Before	NGS	Retrospective	EDTA
	*TP53*	TNBC	OS	37	Before	NGS	Retrospective	EDTA
	*TP53*	TNBC	DFS	30	Before	NGS	Retrospective	EDTA
	*TP53*	ERBB2-positive	DFS	23	Before	NGS	Retrospective	EDTA
Chandarlapaty et al,^6^ 2016	*ESR1*	HR-positive, ERBB2-negative	OS	541	Before	dPCR	Retrospective	EDTA
Chen et al,^7^ 2023	*PIK3CA*	HR-positive, ERBB2-negative	PFS	163	Before	NGS	Prospective	Streck
	*TP53*	HR-positive, ERBB2-negative	PFS	163	Before	NGS	Prospective	Streck
Chin et al,^8^ 2022	*ESR1*	HR-positive, ERBB2-negative	PFS	33	Before	NGS	Retrospective	EDTA
	*PIK3CA*	HR-positive, ERBB2-negative	PFS	33	Before	NGS	Retrospective	EDTA
Clatot et al,^9^ 2016	*ESR1*	All	OS	141	After	dPCR	Retrospective	Heparin
	*ESR1*	All	PFS	140	After	dPCR	Retrospective	Heparin
Cristofanilli et al,^10^ 2016	*PIK3CA*	HR-positive, ERBB2-negative	PFS	395	Before	dPCR	Prospective	NR
Crucitta et al,^11^ 2023	*ESR1*	HR-positive	DFS	28	Before	dPCR	Retrospective	EDTA
Del Re et al,^12^ 2021	*PIK3CA*	All	PFS	30	Before	dPCR	Retrospective	EDTA
Fribbens et al,^13^ 2016 (PALOMA3)	*ESR1*	HR-positive, ERBB2-negative	PFS	360	Before	dPCR	Retrospective	EDTA
Fribbens et al,^13^ 2016 (SoFEA)	*ESR1*	HR-positive, ERBB2-negative	OS	57	Before	dPCR	Retrospective	EDTA
	*ESR1*	HR-positive, ERBB2-negative	PFS	57	Before	dPCR	Retrospective	EDTA
Fribbens et al,^14^ 2018	*KRAS*	All	OS	113	Before	dPCR	Prospective	EDTA
	*KRAS*	All	PFS	113	Before	dPCR	Prospective	EDTA
Fuentes-Antrás et al,^15^ 2023	*PIK3CA*	HR-positive, ERBB2-negative	PFS	75	Before	NGS and dPCR	Prospective	EDTA
	*TP53*	HR-positive, ERBB2-negative	PFS	54	Before	NGS and dPCR	Prospective	EDTA
Guan et al,^16^ 2023	*ERBB2*	All	OS	42	Before	NGS	Prospective	NR
	*PIK3CA*	All	OS	42	Before	NGS	Prospective	NR
	*PIK3CA*	All	PFS	42	Before	NGS	Prospective	NR
	*ERBB2*	All	PFS	42	Before	NGS	Prospective	NR
Gyanchandani et al,^17^ 2016	*ESR1*	HR-positive, ERBB2-negative	OS	14	Before	dPCR	Retrospective	Streck
	*ESR1*	HR-positive, ERBB2-negative	PFS	14	Before	dPCR	Retrospective	Streck
Hu et al,^18^ 2021	*TP53*	All	PFS	17	Before	NGS	Prospective	NR
Kingston et al,^19^ 2021	*ESR1*	All	OS	78	Before	NGS	Prospective	Streck
	*MAPK*	All	OS	58	Before	NGS	Prospective	Streck
	*PIK3CA* (single)	All	PFS	71	Before	NGS	Prospective	Streck
	*PIK3CA* (multiple)	All	PFS	56	Before	NGS	Prospective	Streck
Kumar et al,^20^ 2018	*ESR1*, *PIK3CA*, or *TP53*	HR-positive	PFS	58	Before	dPCR	Prospective	Streck
Lee et al,^21^ 2023	*PIK3CA*	ERBB2-positive	OS	89	NR	NGS	Prospective	Streck
	*PIK3CA*	ERBB2-positive	PFS	89	NR	NGS	Prospective	Streck
	*TP53*	ERBB2-positive	OS	89	NR	NGS	Prospective	Streck
	*TP53*	ERBB2-positive	PFS	89	NR	NGS	Prospective	Streck
Li et al,^22^ 2020	*TP53*	All	OS	45	NR	NGS	Retrospective	EDTA
Liu et al,^23^ 2021	*ESR1*	HR-positive, ERBB2-negative	PFS	25	Before	NGS	Prospective	NR
	*PTEN*	HR-positive, ERBB2-negative	PFS	25	Before	NGS	Prospective	NR
	*ESR1*	HR-positive, ERBB2-negative	PFS	62	Before	NGS	Prospective	NR
	*PTEN*	HR-positive, ERBB2-negative	PFS	62	Before	NGS	Prospective	NR
Liu et al,^24^ 2022	*TP53*	All	PFS	45	Before	NGS	Retrospective	Streck
	*TP53*	All	PFS	52	Before	NGS	Retrospective	Streck
Mosele et al,^25^ 2020	*PIK3CA*	HR-positive, ERBB2-negative	OS	260	After	NGS and dPCR	Retrospective	EDTA
Moynahan et al,^26^ 2017	*PIK3CA*	HR-positive, ERBB2-negative	OS	119	Before	dPCR	Retrospective	EDTA
	*PIK3CA*	HR-positive, ERBB2-negative	PFS	171	Before	dPCR	Retrospective	EDTA
O’Leary et al,^27^ 2018	*ESR1*	HR-positive, ERBB2-negative	PFS	151	Before	dPCR	Retrospective	EDTA
Page et al,^28^ 2017	*ESR1*	All	OS	37	Before	dPCR	Prospective	EDTA
Page et al,^29^ 2021	*ESR1*, *PIK3CA*, *TP53*	All	OS	102	NR	NGS	Prospective	EDTA
Pascual et al,^30^ 2023	*TP53*	HR-positive, ERBB2-negative	OS	201	Before	NGS	Prospective	Streck
	*TP53*	HR-positive, ERBB2-negative	PFS	201	Before	NGS	Prospective	Streck
Raimondi et al,^31^ 2021	*KRAS*	HR-positive, ERBB2-negative	PFS	106	Before	dPCR	Prospective	NR
Schiavon et al,^32^ 2015	*ESR1*	All	PFS	45	Before	dPCR	Prospective	EDTA
Sharma et al,^33^ 2021	*PIK3CA*	All	PFS	42	Before	NGS	Prospective	ACD
Spoerke et al,^34^ 2016	*ESR1* (1 variant)	HR-positive, ERBB2-negative	PFS	58	Before	dPCR	Retrospective	EDTA
	*ESR1* (>1 variant)	HR-positive, ERBB2-negative	PFS	52	Before	dPCR	Retrospective	EDTA
Tang et al,^35^ 2022	*PIK3CA*	HR-positive, ERBB2-negative	PFS	104	After	NGS	Prospective	Streck
	*FGFR*	HR-positive, ERBB2-negative	PFS	104	After	NGS	Prospective	Streck
	*ESR1* and *GATA3*	HR-positive, ERBB2-negative	PFS	104	After	NGS	Prospective	Streck
Tsuji et al,^36^ 2022	*ESR1*	HR-positive, ERBB2-negative	PFS	28	Before	NGS	Prospective	EDTA
	*PIK3CA*	HR-positive, ERBB2-negative	PFS	28	Before	NGS	Prospective	EDTA
Wang et al,^37^ 2023	*FGFR*	HR-positive, ERBB2-negative	DFS	48	Before	NGS	Prospective	Streck
Yi et al,^38^ 2020	*ERBB2*	All	PFS	40	Before	NGS	Prospective	Streck
	*PIK3CA*	All	PFS	10	Before	NGS	Prospective	Streck
Yi et al,^39^ 2020	*PIK3CA*	All	PFS	20	Before	NGS	Retrospective	Streck
Yi et al,^40^ 2020	*TP53*	All	DFS	444	Before	NGS	Retrospective	Streck
Zhang et al,^41^ 2022	*TP53*	All	DFS	35	Before	NGS	Retrospective	EDTA
	*CTCF*, *GNAS*	All	DFS	35	Before	NGS	Retrospective	EDTA
	*TOP1*	All	DFS	35	Before	NGS	Retrospective	EDTA
	*NOTCH2*	All	DFS	35	Before	NGS	Retrospective	EDTA

Abbreviations: DFS, disease-free survival; dPCR, digital polymerase chain reaction; HR, hormone receptor; NGS, next-generation sequencing; NR, not reported; OS, overall survival; PFS, progression-free survival; Streck, cell-free DNA blood collection; TNBC, triple-negative breast cancer.

^a^ Some studies featured multiple survival analyses, each distinct in terms of survival outcomes, detected variants, or breast cancer subtype. Thus, each analysis was treated as an individual study for a total of 75 studies extracted from 37 articles selected in the systematic review.

### Study and Patient Characteristics

The most common survival outcome reported among the 75 studies was PFS (43 studies [57%]),^7,8,9,10,12,13,14,15,16,17,20,21,23,24,26,27,30,31,32,33,34,35,36,38,39^ followed by OS (21 studies [28%])^5,6,9,13,14,16,17,21,22,25,26,28,29,30^ and DFS (11 studies [15%]).^5,11,37,40,41^ Breast cancer subtype of the patient populations differed among studies. Hormone receptor (HR)–positive, ERBB2 (formerly HER2)–negative tumors were analyzed in 34 studies (45%),^5,6,7,8,10,13,15,17,23,25,26,27,30,31,34,35,36,37^ while other studies did not restrict the breast cancer subtype in their analysis and included all subtypes (31 studies [41%]).^5,9,12,14,16,18,19,22,24,28,29,32,33,38,39,40,41^ The least represented subtypes were ERBB2-positive (6 studies [8%]),^5,21^ HR-positive with ERBB2 status not described (2 studies [3%]),^11,20^ and triple-negative breast cancer (TNBC) (2 studies [3%]).^5^

The methods for ctDNA detection also differed among studies, with researchers analyzing plasma ctDNA alterations at baseline by either digital polymerase chain reaction (dPCR) (22 studies 29%)^6,9,10,11,12,13,14,17,20,26,27,28,31,32,34^ or next-generation sequencing (NGS) (50 studies [67%])^5,7,8,16,18,19,21,22,23,24,29,30,33,35,36,37,38,39,40,41^ or a combination of both (3 studies [4%]).^15,25^ All studies that performed NGS used targeted sequencing, except for 1 study that used whole-exome sequencing.^36^ For studies that performed NGS, those reporting survival associated with a single alteration were included.^5,7,8,16,18,19,21,22,23,24,29,30,33,35,36,37,38,39,40,41^ Studies using a variant-agnostic approach by stratifying survival of patients by ctDNA positivity or negativity were excluded.^42,43,44,45,46,47,48^ The most common alterations identified were *TP53* (20 studies [27%]),^5,7,15,18,21,22,24,30,40,41^ *ESR1* (19 studies [25%]),^6,8,9,11,13,17,19,23,27,28,32,34,36^ and *PIK3CA* (19 studies [25%]).^7,8,10,12,15,16,19,21,25,26,33,35,36,38,39^ Two studies (3%)^20,29^ combined patients with either *ESR1*, *TP53*, or *PIK3CA* alterations into their survival analysis. Alterations in *CTCF*,^41^ *ERBB2*,^16,38^ *FGFR*,^35,37^ *GATA3*,^35^ *KRAS*,^14,31^ *MAPK*,^19^ *NOTCH2*,^41^ *PTEN*,^23^ and *TOP1*^41^ were also reported (15 studies [20%]). The distribution of blood tube types across the included studies was as follows: 36 studies (48%) used EDTA tubes,^5,6,8,11,12,13,14,15,22,25,26,27,28,29,32,34,36,41^ 25 (33%) used cfDNA blood-collection (Streck) tubes,^7,17,19,20,21,24,30,35,37,38,39,40^ and 14 (19%) used heparin^9^ or ACD tubes^33^ or did not report the blood tube used.^10,16,18,23,31^ For cfDNA extraction, the majority of studies (67 [89%]) used column-based DNA extraction kits.^5,6,7,9,10,11,12,13,15,16,17,18,19,20,21,22,23,24,26,27,28,31,32,33,34,35,36,37,38,39,40,41^ The remaining 8 (11%) studies used various magnetic bead extraction kits.^8,14,25,29,30^

Other differences were observed in the overall study design among the included studies, such as the timing of the blood draw or the objective of the study (primary vs secondary ctDNA analysis). These differences were accounted for in our meta-regression.

### Association of ctDNA Detection With Survival

First, we investigated the association between ctDNA detection and subsequent survival outcomes. Upon synthesizing findings from all 75 individual studies, the identification of specific alterations in ctDNA was significantly associated with reduced survival (hazard ratio, 1.40; 95% CI, 1.22-1.58; *P* < .001) (eFigure 1 in Supplement 1). Notably, there was statistically significant heterogeneity among these studies (*I*^2^ = 62.10%). Subsequently, the significance persisted on subgroup analysis for each survival outcome (OS: hazard ratio, 1.44 [95% CI, 1.24-1.65; *P* < .001]; PFS: hazard ratio, 1.31 [95% CI, 1.07-1.55; *P* < .001]; DFS: hazard ratio, 1.56 [95% CI, 1.22-1.89; *P* < .001]). All subgroup analyses are summarized in Table 2.

**Table 2.** Summary of Subgroup Analyses

Variable	Hazard ratio (95% CI)	*P* value	*I*^2^, %
Total	1.40 (1.22-1.58)	<.001	62.10
Survival outcome			
OS	1.44 (1.24-1.65)	<.001	7.20
PFS	1.31 (1.07-1.55)	<.001	68.77
DFS	1.56 (1.22-1.89)	<.001	12.78
Breast cancer subtype			
All	1.29 (0.98-1.59)	<.001	60.09
HR-positive/ERBB2 (formerly HER2)–negative	1.38 (1.15-1.61)	<.001	65.16
Other	2.05 (1.49-2.60)	<.001	3.10
Alteration			
* TP53*	1.58 (1.34-1.81)	<.001	12.36
* ESR1*	1.28 (0.96-1.60)	<.001	54.27
* PIK3CA*	1.19 (0.85-1.53)	<.001	80.37
Other	1.82 (1.26-2.39)	<.001	29.52
Study design			
Prospective	1.48 (1.15-1.80)	<.001	71.08
Retrospective	1.37 (1.17-1.56)	<.001	43.72
ctDNA detection method			
NGS	1.48 (1.22-1.74)	<.001	63.56
dPCR	1.28 (1.05-1.50)	<.001	53.92
Blood collection tube			
EDTA	1.40 (1.18-1.63)	<.001	52.85
Streck	1.41 (1.07-1.74)	<.001	71.67
Other	1.40 (1.22-1.58)	<.001	55.14

Abbreviations: ctDNA, circulating tumor DNA; DFS, disease-free survival; dPCR, digital polymerase chain reaction; HR, hormone receptor; NGS, next-generation sequencing; OS, overall survival; PFS, progression-free survival; Streck, cell-free DNA blood collection tube.

Next, we evaluated the strength of the association between ctDNA detection and survival across various breast cancer subtypes. In a subgroup analysis, we found that ctDNA alteration detection in patients with HR-positive, ERBB2-negative breast cancer was associated with reduced survival (hazard ratio, 1.38; 95% CI, 1.15-1.61; *P* < .001) (eFigure 2 in Supplement 1). Combining the less common subtypes examined in the included publications (TNBC, HR-positive, and ERBB2-positive), we identified a consistent pattern of reduced survival in patients with ctDNA alterations (hazard ratio, 2.05; 95% CI, 1.49-2.60; *P* < .001). While studies encompassing all subtypes indicated an association with worse survival, the effect was borderline (hazard ratio, 1.29; 95% CI, 0.98-1.59; *P* < .001).

Given the diverse variant landscape of MBC, we evaluated the association between detection of specific ctDNA alterations and patient survival. We found that *TP53* and *ESR1* alterations were linked to a significantly shorter survival (hazard ratio, 1.58 [95% CI, 1.34-1.81] and 1.28 [95% CI, 0.96-1.60], respectively; *P* < .001) (eFigure 3 in Supplement 1). Furthermore, there was an association between diminished survival and alterations less frequently detected in breast cancer, encompassing *CTCF*, *ERBB2*, *FGFR*, *GATA3*, *KRAS*, *MAPK*, *NOTCH2*, *PTEN*, and *TOP1* (hazard ratio, 1.82; 95% CI, 1.26-2.39; *P* < .001). Interestingly, this pattern was not evident in patients with ctDNA alterations in *PIK3CA* (hazard ratio, 1.19; 95% CI, 0.85-1.53).

Subsequently, we investigated whether variations in the study design characteristics among the included studies were associated with the prognostic value of ctDNA. Reduced survival was observed in both prospective (hazard ratio, 1.48; 95% CI, 1.15-1.80; *P* < .001) and retrospective (hazard ratio, 1.37; 95% CI, 1.17-1.56; *P* < .001) studies (eFigure 4 in Supplement 1). Detection of ctDNA by either NGS (hazard ratio, 1.48; 95% CI, 1.22-1.74; *P* < .001) or dPCR (hazard ratio, 1.28; 95% CI, 1.05-1.50; *P* < .001) was associated with reduced survival (eFigure 5 in Supplement 1). Shorter survival was also observed regardless of the blood tube used, with both Streck and EDTA showing similar outcomes (hazard ratios, 1.41 [95% CI, 1.07-1.74; *P* < .001] and 1.40 [95% CI, 1.18-1.63; *P* < .001], respectively) (eFigure 6 in Supplement 1).

To assess publication bias, we generated a funnel plot. Based on the nonparametric Begg test of rank correlation, publication bias was not significant (eFigure 7 in Supplement 1).

## Discussion

In this systematic review and meta-analysis, detection of specific alterations in ctDNA at baseline was associated with worse survival outcomes in patients with MBC. Subgroup analysis by ctDNA showed that *TP53* and *ESR1* alterations, common driver and treatment resistance events in breast cancer, were significantly associated with worse survival. *PIK3CA* alterations did not show an association with worse survival, however. Our primary finding is consistent with a previously published meta-analysis by Cullinane et al,^49^ which showed an association between elevated ctDNA levels and shorter DFS and PFS; however, their study included patients with all stages of breast cancer. MBC has added genomic complexity compared with early-stage breast cancer that stems from the innate tumor heterogeneity not only of the primary tumor but also across each metastatic site. As a result, studying patients with MBC remains crucial and pertinent.

Owing to the ability of ctDNA to provide a systemic view of metastatic disease, recent studies have investigated the variant landscape of ctDNA in MBC. In a comprehensive study involving 255 patients with MBC, Davis et al^50^ performed NGS on cfDNA, identifying the prevalent ctDNA alterations in distinct breast cancer subtypes. Notably, in patients with HR-positive breast cancer, recurrent alterations were identified in *PIK3CA*, *ESR1*, and *TP53*. Patients with ERBB2-positive MBC exhibited prominent alterations in *TP53*, *PIK3CA*, and *ERBB2*, while those with TNBC harbored alterations in *TP53* and *PIK3CA*. Other commonly altered genes included *MYC*, *EGFR*, *FGFR1*, *CCNE1*, *NF1*, and *ARID1A*. In our meta-analysis, we found that a majority (77%) of the included studies focused on analyzing these hotspot genes. Although worse survival was associated with *ESR1* and not *PIK3CA* alterations, these data shed light on potential avenues for tailored therapeutic interventions in the pursuit of more effective and personalized treatment strategies for patients with MBC.

In our study, we incorporated the results of ctDNA extracted from baseline plasma samples. Defining a baseline sample in the context of metastatic disease is often challenging due to variations in when patients are initially diagnosed with MBC. Approximately 30% of patients diagnosed with early-stage disease progress to metastasis, while approximately 6% are initially diagnosed with metastatic disease.^1^ This distinction is crucial when analyzing ctDNA due to the effect treatments can have on the variant profiles of tumors. For instance, patients with estrogen receptor–positive breast cancer undergoing endocrine therapy may develop treatment resistance due to alterations in *ESR1*, ultimately reducing treatment efficacy.^32,51^ These factors are also important in terms of the approval process of ctDNA tests for clinical use. In 2023, the American Society of Clinical Oncology recommended using liquid biopsy and ctDNA to inform treatment decisions for patients with advanced estrogen receptor–positive, ERBB2-negative breast cancer who experience disease progression after at least 1 line of endocrine therapy.^52^ We conducted an additional subgroup analysis by breast cancer subtype of the 19 studies reporting the survival of patients with *ESR1*.^6,8,9,11,13,17,19,23,27,28,32,34,36^ Interestingly, we did not observe a significant association between the presence of *ESR1* and survival in patients with HR-positive, ERBB2-negative MBC. The lack of significance may be a result of both intra- and interstudy heterogeneity of patient populations. While some studies in our analysis restricted the number of prior treatments received at the time of enrollment, many included patients who received between 0 and 4 lines of treatment at the time of blood collection. Given that *ESR1* alterations are typically linked with treatment resistance and are found very infrequently (approximately 3%) in primary breast cancers,^61^ the ratio of treatment-naive to treatment-exposed patients may have skewed our analysis. We lacked access to patient data for stratification in our analysis. The recent American Society of Clinical Oncology guidelines approve ctDNA tests for specific indications.^52^ Therefore, to enhance liquid biopsy’s clinical integration, increased data transparency and reporting of standardized patient characteristics are imperative. With ctDNA being a limited and valuable resource, data from large, prospective studies could be leveraged for secondary analyses, such as this meta-analysis, to generate significant results to advance the field of liquid biopsy.

The analysis of ctDNA presents a challenge due to its small fraction compared with total cfDNA in a plasma sample, making the choice of blood collection tube crucial. In recent years, several cfDNA stabilizing tubes containing solutions that prevent cfDNA degradation and white blood cell lysis, thereby reducing genomic DNA (gDNA) contamination, have been produced. Additionally, these tubes offer the convenience of room temperature storage for an extended duration (up to 14 days), unlike EDTA tubes, which require processing within a few hours. Determining the purity of a cfDNA sample is important because the presence of gDNA in a plasma sample dilutes the fraction of ctDNA and cfDNA, thereby diminishing the sensitivity of downstream analysis and distorting measures such as variant allele fraction.^53,54^ In a study by Diaz et al,^55^ blood samples from various cancer types were analyzed using either EDTA or Streck tubes. Interestingly, comparable levels of cfDNA yield and gDNA contamination were observed when EDTA and Streck tubes were stored for 3 hours and 6 days, respectively. In our meta-analysis, despite differences in blood collection tubes used among studies, our analysis did not reveal significant differences in the association between ctDNA and survival across groups. Nonetheless, it is imperative for investigators to remain vigilant while performing and documenting blood collection and processing protocols, as minor deviations may considerably influence interpretation of the results.

The choice of cfDNA extraction method is important in ctDNA studies given reported disparities in cfDNA yield and composition across commonly used kits. Bronkhorst et al^56^ conducted a comparative analysis using cell culture supernatant, examining 6 distinct cfDNA extraction kits. Notably, column-based methods showed superior cfDNA yield, albeit with a tendency to capture larger DNA fragments. Conversely, magnetic bead–based methods yielded less cfDNA but exhibited a bias toward recovering shorter DNA fragments. In our meta-analysis, there was a high level of consensus among studies regarding both cfDNA extraction methods, with a majority of studies using column-based methods. While we did not conduct a subgroup analysis, there appears to be a growing trend toward standardized cfDNA extraction protocols in MBC. This trend underscores the need for ongoing refinement in cfDNA extraction methodologies to guarantee robust and reproducible results in ctDNA studies.

In examining the study design, another crucial aspect we investigated was the method used for ctDNA detection. A significant proportion of studies (67%) opted for NGS despite its reduced sensitivity compared with dPCR. Next-generation sequencing offers distinct advantages, particularly in the context of breast cancer and other metastatic cancers, by allowing a tumor-agnostic approach for cfDNA analysis. This approach permits the identification of variants without the need for molecular profiling of tumor tissue, offering a systemic view of the disease by capturing the molecular profiles of both primary and metastatic tumors. However, false-positive results may arise due to clonal hematopoiesis of indeterminate potential.^57^ While these variants can be filtered to improve sensitivity, several studies included in our meta-analysis did not describe their bioinformatics pipeline in detail, making it difficult to ascertain whether these variants were removed. On the contrary, dPCR uses a tumor-informed approach, necessitating specific information about the tumor to design variant-specific ctDNA assays. While dPCR boasts higher sensitivity compared with NGS, its reliance on tissue biopsies, primarily from primary tumors, may overlook the tumor heterogeneity observed at other metastatic sites. To incorporate studies using both NGS and dPCR to detect ctDNA, our meta-analysis specifically focused on those examining survival associated with the detection of a single variant in cfDNA at baseline. Our findings revealed that both dPCR and NGS were associated with worse survival outcomes in patients with MBC. Hence, an optimal strategy in a clinical setting might involve using a combination of NGS and dPCR throughout a patient’s treatment course. This integrated strategy would improve ctDNA surveillance, ensuring a more comprehensive understanding and timely response to changes in the molecular landscape.

### Limitations

This study has some limitations. Although we included 37 articles in our meta-analysis, several relevant studies were excluded due to lack of data availability. Many studies were missing key information, such as sample size of each group in the survival analysis, hazard ratios, and/or confidence intervals. Additionally, we encountered several studies that stratified patient survival using metrics such as *z* score^58^ or genomic instability number.^59^ We also identified several articles that established an association between ctDNA levels and treatment resistance and/or response.^60^ However, these articles were beyond the scope of our research question. None of the included studies enrolled male participants, which is a limitation of our meta-analysis as this population remains important to, yet often underrepresented in, breast cancer research.

## Conclusions

In this systematic review and meta-analysis, we highlight the prognostic value of single ctDNA alteration detection in patients with MBC. While prognostic biomarkers are not yet clinically actionable, the field is moving in this direction. By identifying the associations between prognosis and ctDNA, ctDNA can subsequently be correlated with specific therapeutic decisions in the future. These findings may guide the design of future clinical trials and prospective studies that will ultimately benefit patients with MBC.

## Notes

Supplement 1. **eFigure 1.** Association Between Detectable ctDNA Alterations and Reduced Survival **eFigure 2.** Subgroup Analysis of Breast Cancer Subtypes **eFigure 3.** Subgroup Analysis of ctDNA Variants **eFigure 4.** Subgroup Analysis of Study Design **eFigure 5.** Subgroup Analysis of ctDNA Detection Methods **eFigure 6.** Subgroup Analysis of Blood Collection Tube Used **eFigure 7.** Funnel Plot to Assess Publication Bias

Supplement 2. **eTable 1.** Detailed Search Strategy for Each Included Database **eTable 2.** Newcastle-Ottawa Score (NOS) Ratings for Included Studies

Supplement 3.
Data Sharing Statement
