# Supplementary material for: Circulating Tumor DNA and Survival in Metastatic Breast Cancer: A Systematic Review and Meta-Analysis
Source: JAMA Netw Open. 2024 Sep 5;7(9):e2431722. doi: 10.1001/jamanetworkopen.2024.31722 (PMC11378006; doi:10.1001/jamanetworkopen.2024.31722)
Supplement: Supplement 1. — eFigure 1. Association Between Detectable ctDNA Alterations and Reduced Survival eFigure 2. Subgroup Analysis of Breast Cancer Subtypes eFigure 3. Subgroup Analysis of ctDNA Variants eFigure 4. Subgroup Analysis of Study Design eFigure 5. Subgroup Analysis of ctDNA Detection Methods eFigure 6. Subgroup Analysis of Blood Collection Tube Used eFigure 7. Funnel Plot to Assess Publication Bias [file jamanetwopen-e2431722-s001.pdf]

Supplemental Online Content

Dickinson K, Sharma A, Agnihotram RKV, et al. Circulating tumor DNA and survival in metastatic breast cancer: a systematic review and meta-analysis. *JAMA Netw Open*. 2024;7(9):e2431722. doi:10.1001/jamanetworkopen.2024.31722

eFigure 1. Association Between Detectable ctDNA Alterations and Reduced Survival

eFigure 2. Subgroup Analysis of Breast Cancer Subtypes

eFigure 3. Subgroup Analysis of ctDNA Alterations

eFigure 4. Subgroup Analysis of Study Design

eFigure 5. Subgroup Analysis of ctDNA Detection Methods

eFigure 6. Subgroup Analysis of Blood Collection Tube Used

eFigure 7. Funnel Plot to Assess Publication Bias

This supplemental material has been provided by the authors to give readers additional information about their work.

© 2024 Dickinson K et al. *JAMA Network Open*.

eFigure 1. Association Between Detectable ctDNA Alterations and Reduced Survival. Forest plot of pooled meta-analysis including 75 studies.

Source	Mutation	Breast cancer subtype	Outcome tested	HR [95% CI]	Weight (%)
Bai et al,[5] 2021	*TP53*	All	OS	1.99 [0.97, 3.01]	1.62
Bai et al,[5] 2021	*TP53*	All	DFS	1.89 [1.19, 2.59]	2.27
Bai et al,[5] 2021	*TP53*	HER2+	OS	2.47 [-0.49, 5.43]	0.33
Bai et al,[5] 2021	*TP53*	HER2+	DFS	1.10 [0.04, 2.16]	1.56
Bai et al,[5] 2021	*TP53*	HR+, HER2-	OS	1.20 [0.28, 2.12]	1.80
Bai et al,[5] 2021	*TP53*	HR+, HER2-	DFS	1.57 [0.79, 2.35]	2.08
Bai et al,[5] 2021	*TP53*	TNBC	OS	2.42 [-0.83, 5.67]	0.28
Bai et al,[5] 2021	*TP53*	TNBC	DFS	2.17 [0.20, 4.14]	0.66
Chandarlapaty et al,[6] 2016	*ESR1*	HR+, HER2-	OS	1.62 [1.25, 1.99]	3.04
Chen et al,[7] 2023	*PIK3CA*	HR+, HER2-	PFS	1.74 [1.01, 2.47]	2.20
Chen et al,[7] 2023	*TP53*	HR+, HER2-	PFS	1.64 [0.81, 2.47]	1.99
Chin et al,[8] 2022	*ESR1*	HR+, HER2-	PFS	5.25 [-3.25, 13.54]	0.05
Chin et al,[8] 2022	*PIK3CA*	HR+, HER2-	PFS	1.16 [-0.27, 2.59]	1.07
Clatot et al,[9] 2016	*ESR1*	All	OS	1.90 [0.95, 2.85]	1.75
Clatot et al,[9] 2016	*ESR1*	All	PFS	1.70 [1.00, 2.40]	2.27
Cristofanilli et al,[10] 2016	*PIK3CA*	HR+, HER2-	PFS	1.26 [0.89, 1.63]	3.04
Crucitta et al,[11] 2023	*ESR1*	HR+	DFS	3.18 [0.03, 6.33]	0.29
Del Re et al,[12] 2020	*PIK3CA*	All	PFS	2.93 [0.07, 5.79]	0.35
Fribbens et al,[13] 2016 (SoFEA)	*ESR1*	HR+, HER2-	OS	1.65 [0.37, 2.93]	1.24
Fribbens et al,[14] 2018	*KRAS*	All	OS	1.16 [0.48, 1.84]	2.31
Fribbens et al,[14] 2018	*KRAS*	All	PFS	1.04 [0.53, 1.55]	2.72
Fribbens et al,[13] 2016 (PALOMA3)	*ESR1*	HR+, HER2-	OS	1.46 [0.98, 1.94]	2.79
Fribbens et al,[13] 2016 (SoFEA)	*ESR1*	HR+, HER2-	PFS	2.12 [0.81, 3.43]	1.20
Fuentes-Antrás et al,[15] 2023	*PIK3CA*	HR+, HER2-	PFS	2.14 [0.92, 3.36]	1.32
Fuentes-Antrás et al,[15] 2023	*TP53*	HR+, HER2-	PFS	2.40 [-0.17, 4.97]	0.42
Guan et al,[16] 2023	*ERBB2*	All	OS	10.10 [-7.04, 27.24]	0.01
Guan et al,[16] 2023	*PIK3CA*	All	OS	1.58 [0.18, 2.98]	1.10
Guan et al,[16] 2023	*PIK3CA*	All	PFS	2.99 [0.08, 5.90]	0.34
Guan et al,[16] 2023	*ERBB2*	All	PFS	4.43 [-1.80, 10.66]	0.08
Gyanchandani et al,[17] 2017	*ESR1*	HR+, HER2-	OS	0.32 [-0.72, 1.36]	1.60
Gyanchandani et al,[17] 2017	*ESR1*	HR+, HER2-	PFS	0.25 [-0.19, 0.69]	2.87
Hu et al,[18] 2021	*TP53*	All	PFS	3.57 [-3.09, 10.23]	0.07
Kingston et al,[19] 2021	*ESR1*	All	OS	0.82 [0.18, 1.46]	2.40
Kingston et al,[19] 2021	*MAPK*	All	OS	1.65 [-0.51, 3.81]	0.57
Kingston et al,[19] 2021	*PIK3CA* (single)	All	PFS	0.71 [0.31, 1.11]	2.96
Kingston et al,[19] 2021	*PIK3CA* (multiple)	All	PFS	3.15 [-2.07, 8.37]	0.11
Kumar et al,[20] 2018	*ESR1, PIK3CA, or TP53*	HR+	PFS	3.80 [0.95, 6.65]	0.35
Lee et al,[21] 2023	*PIK3CA*	HER2+	OS	2.86 [0.13, 5.59]	0.38
Lee et al,[21] 2023	*PIK3CA*	HER2+	PFS	2.07 [0.82, 3.32]	1.28
Lee et al,[21] 2023	*TP53*	HER2+	OS	2.61 [0.38, 4.84]	0.54
Lee et al,[21] 2023	*TP53*	HER2+	PFS	2.17 [1.11, 3.23]	1.56
Li et al,[22] 2020	*TP53*	All	OS	4.31 [-1.45, 10.07]	0.09
Liu et al,[23] 2021	*ESR1*	HR+, HER2-	PFS	1.20 [-1.58, 3.98]	0.37
Liu et al,[24] 2022	*TP53*	All	PFS	1.00 [0.23, 1.77]	2.10
Liu et al,[23] 2021	*PTEN*	HR+, HER2-	PFS	1.28 [-3.77, 6.33]	0.12
Liu et al,[24] 2022	*TP53*	All	PFS	1.19 [0.31, 2.07]	1.88
Liu et al,[23] 2021	*ESR1*	HR+, HER2-	PFS	2.40 [0.15, 4.65]	0.53
Liu et al,[23] 2021	*PTEN*	HR+, HER2-	PFS	3.50 [-1.89, 8.89]	0.11
Mosele et al,[25] 2020	*PIK3CA*	HR+, HER2-	OS	1.44 [0.94, 1.94]	2.73
Moynahan et al,[26] 2017	*PIK3CA*	HR+, HER2-	OS	1.32 [0.98, 1.66]	3.09
Moynahan et al,[26] 2017	*PIK3CA*	HR+, HER2-	PFS	1.26 [0.98, 1.54]	3.21
O'Leary et al,[27] 2018	*ESR1*	HR+, HER2-	PFS	1.58 [0.88, 2.28]	2.26
Page et al,[28] 2017	*ESR1*	All	OS	25.61 [-43.69, 94.91]	0.00
Page et al,[29] 2021	*ESR1/PIK3CA/TP53*	All	OS	3.46 [1.19, 5.73]	0.52
Pascual et al,[30] 2023	*TP53*	HR+, HER2-	OS	2.89 [1.36, 4.42]	0.97
Pascual et al,[30] 2023	*TP53*	HR+, HER2-	PFS	1.84 [1.17, 2.51]	2.33
Raimondi et al,[31] 2021	*KRAS*	HR+, HER2-	PFS	20.75 [3.07, 38.43]	0.01
Schiavon et al,[32] 2015	*ESR1*	All	PFS	3.10 [-7.50, 13.70]	0.03
Sharma et al,[33] 2021	*PIK3CA*	All	PFS	0.44 [0.08, 0.80]	3.06
Spoerke et al,[34] 2016	*ESR1* (1 mutation)	HR+, HER2-	PFS	0.89 [0.29, 1.49]	2.50
Spoerke et al,[34] 2016	*ESR1* (>1 mutation)	HR+, HER2-	PFS	1.39 [0.29, 2.49]	1.50
Tang et al,[35] 2022	*PIK3CA*	HR+, HER2-	PFS	2.04 [0.77, 3.31]	1.26
Tang et al,[35] 2022	*FGFR*	HR+, HER2-	PFS	1.90 [0.62, 3.18]	1.24
Tang et al,[35] 2022	*ESR1 + GATA3*	HR+, HER2-	PFS	1.87 [0.91, 2.83]	1.73
Tsiji et al,[36] 2022	*ESR1*	HR+, HER2-	PFS	1.00 [0.00, 2.00]	1.66
Tsiji et al,[36] 2022	*PIK3CA*	HR+, HER2-	PFS	0.20 [-0.07, 0.47]	3.24
Wang et al,[37] 2023	*FGFR*	HR+, HER2-	DFS	2.33 [0.55, 4.11]	0.78
Yi et al,[38] 2020	*PIK3CA*	All	PFS	1.20 [-0.31, 2.71]	0.99
Yi et al,[38] 2020	*TP53*	All	PFS	1.32 [1.06, 1.58]	3.25
Yi et al,[39] 2020	*ERBB2*	All	PFS	3.37 [-0.09, 6.83]	0.25
Yi et al,[40] 2020	*PIK3CA*	All	DFS	0.18 [-0.27, 0.63]	2.86
Zhang et al,[41] 2022	*TP53*	All	DFS	3.14 [0.23, 6.05]	0.34
Zhang et al,[41] 2022	*CTCF/GNAS*	All	DFS	7.23 [-6.57, 21.03]	0.02
Zhang et al,[41] 2022	*TOP1*	All	DFS	7.21 [-6.94, 21.36]	0.02
Zhang et al,[41] 2022	*NOTCH2*	All	DFS	3.44 [-2.13, 9.01]	0.10
**Total**				**1.40 [1.22, 1.58]**	**100**

Heterogeneity: $\tau^2 = 0.24$, $I^2 = 62.10\%$, $H^2 = 2.64$

Test of overall effect: $z = 15.42$, $p = <0.001$

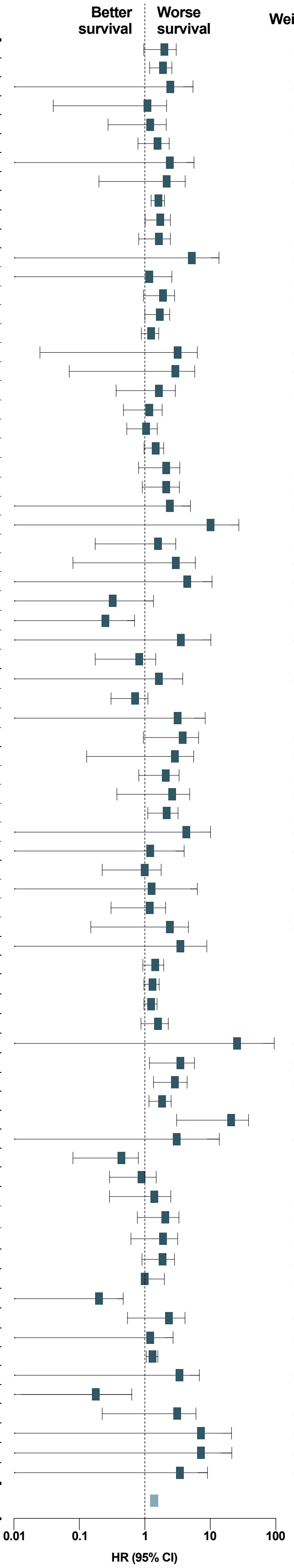

© 2024 Dickinson K et al. *JAMA Network Open*.

eFigure 2. Subgroup Analysis of Breast Cancer Subtypes

A. All

Source	Mutation	Outcome tested	HR [95% CI]
Bai et al,[5] 2021	*TP53*	OS	1.99 [0.97, 3.01]
Bai et al,[5] 2021	*TP53*	DFS	1.89 [1.19, 2.59]
Clatot et al,[9] 2016	*ESR1*	OS	1.90 [0.95, 2.85]
Clatot et al,[9] 2016	*ESR1*	PFS	1.70 [1.00, 2.40]
Del Re et al,[12] 2020	*PIK3CA*	PFS	2.93 [0.07, 5.79]
Fribbens et al,[14] 2018	*KRAS*	OS	1.16 [0.48, 1.84]
Fribbens et al,[14] 2018	*KRAS*	PFS	1.04 [0.53, 1.55]
Guan et al,[16] 2023	*ERBB2*	OS	10.10 [-7.04, 27.24]
Guan et al,[16] 2023	*PIK3CA*	OS	1.58 [0.18, 2.98]
Guan et al,[16] 2023	*PIK3CA*	PFS	2.99 [0.08, 5.90]
Guan et al,[16] 2023	*ERBB2*	PFS	4.43 [-1.80, 10.66]
Hu et al,[18] 2021	*TP53*	PFS	3.57 [-3.09, 10.23]
Kingston et al,[19] 2021	*ESR1*	OS	0.82 [0.18, 1.46]
Kingston et al,[19] 2021	*MAPK*	OS	1.65 [-0.51, 3.81]
Kingston et al,[19] 2021	*PIK3CA* (single)	PFS	0.71 [0.31, 1.11]
Kingston et al,[19] 2021	*PIK3CA* (multiple)	PFS	3.15 [-2.07, 8.37]
Li et al,[22] 2020	*TP53*	OS	4.31 [-1.45, 10.07]
Liu et al,[24] 2022	*TP53*	PFS	1.00 [0.23, 1.77]
Liu et al,[24] 2022	*TP53*	PFS	1.19 [0.31, 2.07]
Page et al,[28] 2017	*ESR1*	OS	25.61 [-43.69, 94.91]
Page et al,[29] 2021	*ESR1/PIK3CA/TP53*	OS	3.46 [1.19, 5.73]
Schiavon et al,[32] 2015	*ESR1*	PFS	3.10 [-7.50, 13.70]
Sharma et al,[33] 2021	*PIK3CA*	PFS	0.44 [0.08, 0.80]
Yi et al,[38] 2020	*PIK3CA*	PFS	1.20 [-0.31, 2.71]
Yi et al,[38] 2020	*TP53*	PFS	1.32 [1.06, 1.58]
Yi et al,[39] 2020	*ERBB2*	PFS	3.37 [-0.09, 6.83]
Yi et al,[40] 2020	*PIK3CA*	DFS	0.18 [-0.27, 0.63]
Zhang et al,[41] 2022	*TP53*	DFS	3.14 [0.23, 6.05]
Zhang et al,[41] 2022	*CTCF/GNAS*	DFS	7.23 [-6.57, 21.03]
Zhang et al,[41] 2022	*TOP1*	DFS	7.21 [-6.94, 21.36]
Zhang et al,[41] 2022	*NOTCH2*	DFS	3.44 [-2.13, 9.01]
**Total**			**1.29 [0.98, 1.59]**

Heterogeneity: $\tau^2 = 0.26$, $I^2 = 60.09\%$, $H^2 = 2.51$
Test of overall effect: $z = 8.32$, $p = <0.001$

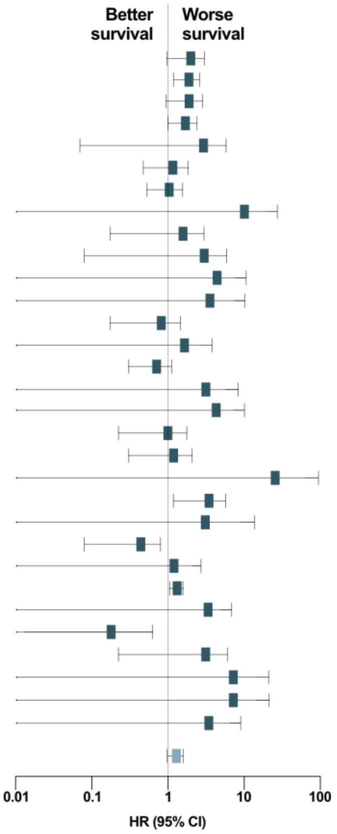

© 2024 Dickinson K et al. *JAMA Network Open*.

B. HR+, HER2-

Source	Mutation	Outcome tested	HR [95% CI]
Bai et al,[5] 2021	*TP53*	OS	1.20 [0.28, 2.12]
Bai et al,[5] 2021	*TP53*	DFS	1.57 [0.79, 2.35]
Chandarlapaty et al,[6] 2016	*ESR1*	OS	1.62 [1.25, 1.99]
Chen et al,[7] 2023	*PIK3CA*	PFS	1.74 [1.01, 2.47]
Chen et al,[7] 2023	*TP53*	PFS	1.64 [0.81, 2.47]
Chin et al,[8] 2022	*ESR1*	PFS	5.25 [-3.25, 13.54]
Chin et al,[8] 2022	*PIK3CA*	PFS	1.16 [-0.27, 2.59]
Cristofanilli et al,[10] 2016	*PIK3CA*	PFS	1.26 [0.89, 1.63]
Fribbens et al,[13] 2016 (SoFEA)	*ESR1*	OS	1.65 [0.37, 2.93]
Fribbens et al,[13] 2016 (PALOMA3)	*ESR1*	OS	1.46 [0.98, 1.94]
Fribbens et al,[13] 2016 (SoFEA)	*ESR1*	PFS	2.12 [0.81, 3.43]
Fuentes-Antrás et al,[15] 2023	*PIK3CA*	PFS	2.14 [0.92, 3.36]
Fuentes-Antrás et al,[15] 2023	*TP53*	PFS	2.40 [-0.17, 4.97]
Gyanchandani et al,[17] 2017	*ESR1*	OS	0.32 [-0.72, 1.36]
Gyanchandani et al,[17] 2017	*ESR1*	PFS	0.25 [-0.19, 0.69]
Liu et al,[23] 2021	*ESR1*	PFS	1.20 [-1.58, 3.98]
Liu et al,[23] 2021	*PTEN*	PFS	1.28 [-3.77, 6.33]
Liu et al,[23] 2021	*ESR1*	PFS	2.40 [0.15, 4.65]
Liu et al,[23] 2021	*PTEN*	PFS	3.50 [-1.89, 8.89]
Mosele et al,[25] 2020	*PIK3CA*	OS	1.44 [0.94, 1.94]
Moynahan et al,[26] 2017	*PIK3CA*	OS	1.32 [0.98, 1.66]
Moynahan et al,[26] 2017	*PIK3CA*	PFS	1.26 [0.98, 1.54]
O'Leary et al,[27] 2018	*ESR1*	PFS	1.58 [0.88, 2.28]
Pascual et al,[30] 2023	*TP53*	OS	2.89 [1.36, 4.42]
Pascual et al,[30] 2023	*TP53*	PFS	1.84 [1.17, 2.51]
Raimondi et al,[31] 2021	*KRAS*	PFS	20.75 [3.07, 38.43]
Spoerke et al,[34] 2016	*ESR1* (1 mutation)	PFS	0.89 [0.29, 1.49]
Spoerke et al,[34] 2016	*ESR1* (>1 mutation)	PFS	1.39 [0.29, 2.49]
Tang et al,[35] 2022	*PIK3CA*	PFS	2.04 [0.77, 3.31]
Tang et al,[35] 2022	*FGFR*	PFS	1.90 [0.62, 3.18]
Tang et al,[35] 2022	*ESR1 + GATA3*	PFS	1.87 [0.91, 2.83]
Tsiji et al,[36] 2022	*ESR1*	PFS	1.00 [0.00, 2.00]
Tsiji et al,[36] 2022	*PIK3CA*	PFS	0.20 [-0.07, 0.47]
Wang et al,[37] 2023	*FGFR*	DFS	2.33 [0.55, 4.11]
**Total**			**1.38 [1.15, 1.61]**

Heterogeneity: τ^2 = 0.21, I^2 = 65.16%, H^2 = 2.87
Test of overall effect: z = 11.85, p = <0.001

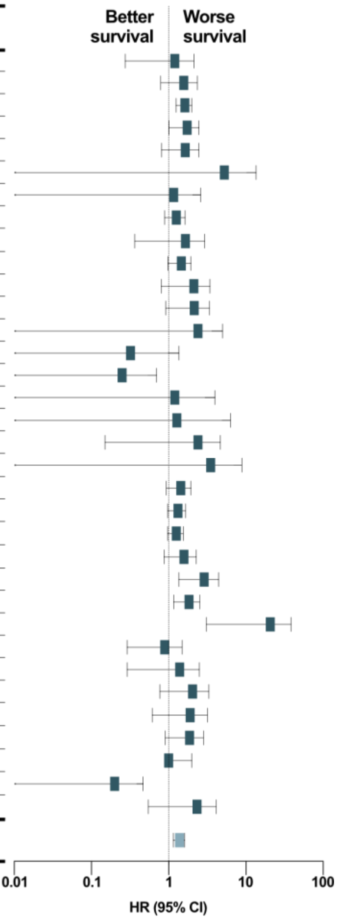

C. Other

Source	Mutation	Outcome tested	HR [95% CI]
Bai et al,[5] 2021	*TP53*	OS	2.47 [-0.49, 5.43]
Bai et al,[5] 2021	*TP53*	DFS	1.10 [0.04, 2.16]
Bai et al,[5] 2021	*TP53*	OS	2.42 [-0.83, 5.67]
Bai et al,[5] 2021	*TP53*	DFS	2.17 [0.20, 4.14]
Crucitta et al,[11] 2023	*TP53*	DFS	3.18 [0.03, 6.33]
Kumar et al,[20] 2018	*ESR1*	PFS	3.80 [0.95, 6.65]
Lee et al,[21] 2023	*PIK3CA*	OS	2.86 [0.13, 5.59]
Lee et al,[21] 2023	*PIK3CA*	PFS	2.07 [0.82, 3.32]
Lee et al,[21] 2023	*ESR1*	OS	2.61 [0.38, 4.84]
Lee et al,[21] 2023	*ESR1*	PFS	2.17 [1.11, 3.23]
**Total**			**2.05 [1.49, 2.60]**

Heterogeneity: τ^2 = 0.03, I^2 = 3.1%, H^2 = 1.03
Test of overall effect: z = 7.21, p = <0.001

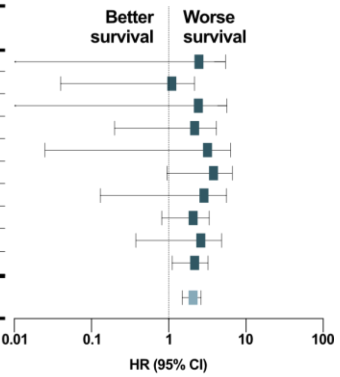

© 2024 Dickinson K et al. *JAMA Network Open*.

eFigure 3. Subgroup Analysis of ctDNA Alterations

A. *TP53*

Source	Breast cancer subtype	Outcome tested	HR [95% CI]
Bai et al,[5] 2021	All	OS	1.99 [0.97, 3.01]
Bai et al,[5] 2021	All	DFS	1.89 [1.19, 2.59]
Bai et al,[5] 2021	HER2+	OS	2.47 [-0.49, 5.43]
Bai et al,[5] 2021	HER2+	DFS	1.10 [0.04, 2.16]
Bai et al,[5] 2021	HR+, HER2-	OS	1.20 [0.28, 2.12]
Bai et al,[5] 2021	HR+, HER2-	DFS	1.57 [0.79, 2.35]
Bai et al,[5] 2021	TNBC	OS	2.42 [-0.83, 5.67]
Bai et al,[5] 2021	TNBC	DFS	2.17 [0.20, 4.14]
Chen et al,[7] 2023	HR+, HER2-	PFS	1.64 [0.81, 2.47]
Fuentes-Antrás et al,[15] 2023	HR+, HER2-	PFS	2.40 [-0.17, 4.97]
Hu et al,[18] 2021	All	PFS	3.57 [-3.09, 10.23]
Lee et al,[21] 2023	HER2+	OS	2.61 [0.38, 4.84]
Lee et al,[21] 2023	HER2+	PFS	2.17 [1.11, 3.23]
Li et al,[22] 2020	All	OS	4.31 [-1.45, 10.07]
Liu et al,[24] 2022	All	PFS	1.00 [0.23, 1.77]
Liu et al,[24] 2022	All	PFS	1.19 [0.31, 2.07]
Pascual et al,[30] 2023	HR+, HER2-	OS	2.89 [1.36, 4.42]
Pascual et al,[30] 2023	HR+, HER2-	PFS	1.84 [1.17, 2.51]
Yi et al,[38] 2020	All	PFS	1.32 [1.06, 1.58]
Zhang et al,[41] 2022	All	DFS	3.14 [0.23, 6.05]
**Total**			**1.58 [1.34, 1.81]**

Heterogeneity: $\tau^2 = 0.03$, $I^2 = 12.36\%$, $H^2 = 1.14$
Test of overall effect: $z = 13.35$, p = <0.001

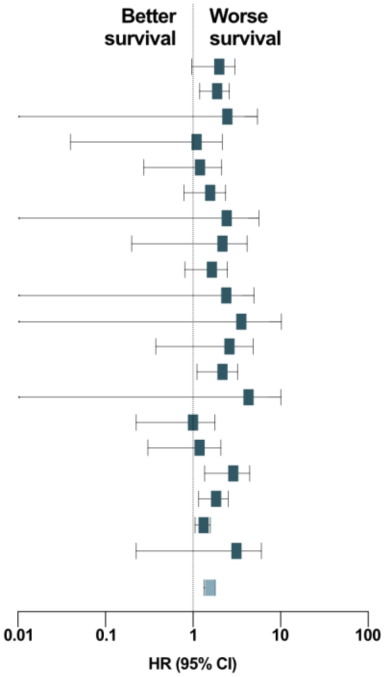

B. *ESR1*

Source	Breast cancer subtype	Outcome tested	HR [95% CI]
Chandarlapaty et al,[6] 2016	HR+, HER2-	OS	1.62 [1.25, 1.99]
Chin et al,[8] 2022	HR+, HER2-	PFS	5.25 [-3.25, 13.54]
Clatot et al,[9] 2016	All	OS	1.90 [0.95, 2.85]
Clatot et al,[9] 2016	All	PFS	1.70 [1.00, 2.40]
Crucitta et al,[11] 2023	HR+	DFS	3.18 [0.03, 6.33]
Fribbens et al,[13] 2016 (SoFEA)	HR+, HER2-	OS	1.65 [0.37, 2.93]
Fribbens et al,[13] 2016 (PALOMA3)	HR+, HER2-	OS	1.46 [0.98, 1.94]
Fribbens et al,[13] 2016 (SoFEA)	HR+, HER2-	PFS	2.12 [0.81, 3.43]
Gyanchandani et al,[17] 2017	HR+, HER2-	OS	0.32 [-0.72, 1.36]
Gyanchandani et al,[17] 2017	HR+, HER2-	PFS	0.25 [-0.19, 0.69]
Kingston et al,[19] 2021	All	OS	0.82 [0.18, 1.46]
Liu et al,[23] 2021	HR+, HER2-	PFS	1.20 [-1.58, 3.98]
Liu et al,[23] 2021	HR+, HER2-	PFS	2.40 [0.15, 4.65]
O'Leary et al,[27] 2018	HR+, HER2-	PFS	1.58 [0.88, 2.28]
Page et al,[28] 2017	All	OS	25.61 [-43.69, 94.91]
Schiavon et al,[32] 2015	All	PFS	3.10 [-7.50, 13.70]
Spoerke et al,[34] 2016	HR+, HER2-	PFS	0.89 [0.29, 1.49]
Spoerke et al,[34] 2016	HR+, HER2-	PFS	1.39 [0.29, 2.49]
Tsiji et al,[36] 2022	HR+, HER2-	PFS	1.00 [0.00, 2.00]
**Total**			**1.28 [0.96, 1.60]**

Heterogeneity: $\tau^2 = 0.20$, $I^2 = 54.27\%$, $H^2 = 2.19$
Test of overall effect: $z = 7.87$, p = <0.001

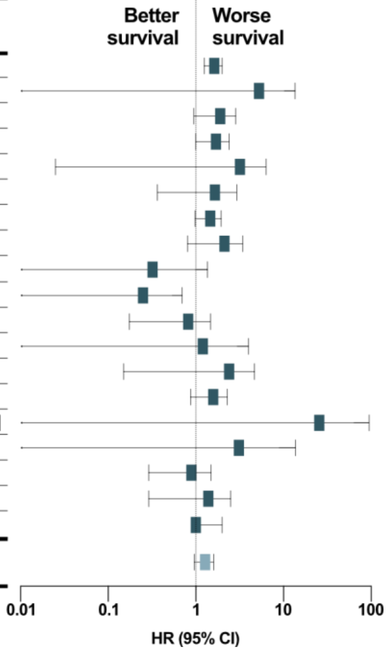

© 2024 Dickinson K et al. *JAMA Network Open*.

C. *PIK3CA*

Source	Breast cancer subtype	Outcome tested	HR [95% CI]
Chen et al,[7] 2023	HR+, HER2-	PFS	1.74 [1.01, 2.47]
Chin et al,[8] 2022	HR+, HER2-	PFS	1.16 [-0.27, 2.59]
Cristofanilli et al,[10] 2016	HR+, HER2-	PFS	1.26 [0.89, 1.63]
Del Re et al,[12] 2020	All	PFS	2.93 [0.07, 5.79]
Fuentes-Antrás et al,[15] 2023	HR+, HER2-	PFS	2.14 [0.92, 3.36]
Guan et al,[16] 2023	All	OS	1.58 [0.18, 2.98]
Guan et al,[16] 2023	All	PFS	2.99 [0.08, 5.90]
Kingston et al,[19] 2021	All	PFS	0.71 [0.31, 1.11]
Kingston et al,[19] 2021	All	PFS	3.15 [-2.07, 8.37]
Lee et al,[21] 2023	HER2+	OS	2.86 [0.13, 5.59]
Lee et al,[21] 2023	HER2+	PFS	2.07 [0.82, 3.32]
Mosele et al,[25] 2020	HR+, HER2-	OS	1.44 [0.94, 1.94]
Moynahan et al,[26] 2017	HR+, HER2-	OS	1.32 [0.98, 1.66]
Moynahan et al,[26] 2017	HR+, HER2-	PFS	1.26 [0.98, 1.54]
Sharma et al,[33] 2021	All	PFS	0.44 [0.08, 0.80]
Tang et al,[35] 2022	HR+, HER2-	PFS	2.04 [0.77, 3.31]
Tsiji et al,[36] 2022	HR+, HER2-	PFS	0.20 [-0.07, 0.47]
Yi et al,[38] 2020	All	PFS	1.20 [-0.31, 2.71]
Yi et al,[40] 2020	All	DFS	0.18 [-0.27, 0.63]
**Total**			**1.19 [0.85, 1.53]**

Heterogeneity: $\tau^2 = 0.31$, $I^2 = 80.37\%$, $H^2 = 5.09$
Test of overall effect: z = 6.96, p = <0.001

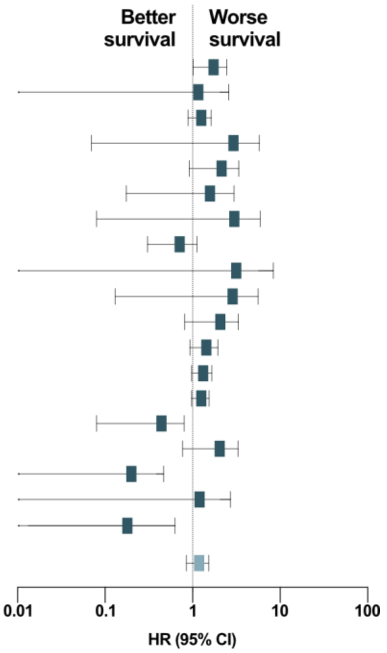

D. Other

Source	Breast cancer subtype	Outcome tested	HR [95% CI]
Fribbens et al,[14] 2018	All	OS	1.16 [0.48, 1.84]
Fribbens et al,[14] 2018	All	PFS	1.04 [0.53, 1.55]
Guan et al,[16] 2023	All	OS	10.10 [-7.04, 27.24]
Guan et al,[16] 2023	All	PFS	4.43 [-1.80, 10.66]
Kingston et al,[19] 2021	All	OS	1.65 [-0.51, 3.81]
Kumar et al,[20] 2018	HR+	PFS	3.80 [0.95, 6.65]
Liu et al,[23] 2021	HR+, HER2-	PFS	1.28 [-3.77, 6.33]
Liu et al,[23] 2021	HR+, HER2-	PFS	3.50 [-1.89, 8.89]
Page et al,[29] 2021	All	OS	3.46 [1.19, 5.73]
Raimondi et al,[31] 2021	HR+, HER2-	PFS	20.75 [3.07, 38.43]
Tang et al,[35] 2022	HR+, HER2-	PFS	1.90 [0.62, 3.18]
Tang et al,[35] 2022	HR+, HER2-	PFS	1.87 [0.91, 2.83]
Wang et al,[37] 2023	HR+, HER2-	DFS	2.33 [0.55, 4.11]
Yi et al,[39] 2020	All	PFS	3.37 [-0.09, 6.83]
Zhang et al,[41] 2022	All	DFS	7.23 [-6.57, 21.03]
Zhang et al,[41] 2022	All	DFS	7.21 [-6.94, 21.36]
Zhang et al,[41] 2022	All	DFS	3.44 [-2.13, 9.01]
**Total**			**1.82 [1.26, 2.39]**

Heterogeneity: $\tau^2 = 0.28$, $I^2 = 29.52\%$, $H^2 = 1.42$
Test of overall effect: z = 6.38, p = <0.001

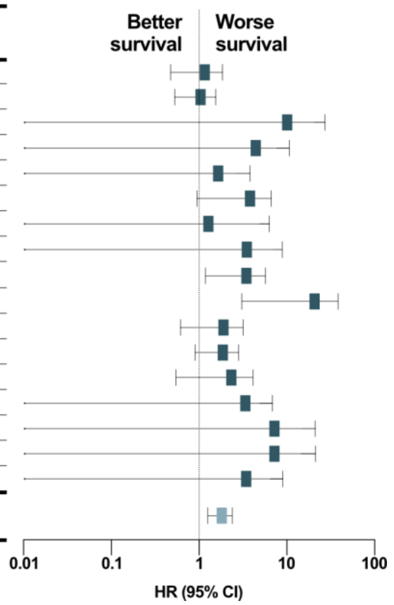

© 2024 Dickinson K et al. *JAMA Network Open*.

eFigure 4. Subgroup Analysis of Study Design

A. Prospective

Source	Mutation	Breast cancer subtype	Outcome tested	HR [95% CI]
Chen et al,[7] 2023	*PIK3CA*	HR+, HER2-	PFS	1.74 [1.01, 2.47]
Chen et al,[7] 2023	*TP53*	HR+, HER2-	PFS	1.64 [0.81, 2.47]
Cristofanilli et al,[10] 2016	*PIK3CA*	HR+, HER2-	PFS	1.26 [0.89, 1.63]
Fribbens et al,[14] 2018	*KRAS*	All	OS	1.16 [0.48, 1.84]
Fribbens et al,[14] 2018	*KRAS*	All	PFS	1.04 [0.53, 1.55]
Guan et al,[16] 2023	*ERBB2*	All	OS	10.10 [-7.04, 27.24]
Guan et al,[16] 2023	*PIK3CA*	All	OS	1.58 [0.18, 2.98]
Guan et al,[16] 2023	*PIK3CA*	All	PFS	2.99 [0.08, 5.90]
Guan et al,[16] 2023	*ERBB2*	All	PFS	4.43 [-1.80, 10.66]
Hu et al,[18] 2021	*TP53*	All	PFS	3.57 [-3.09, 10.23]
Kingston et al,[19] 2021	*ESR1*	All	OS	0.82 [0.18, 1.46]
Kingston et al,[19] 2021	*MAPK*	All	OS	1.65 [-0.51, 3.81]
Kingston et al,[19] 2021	*PIK3CA* (single)	All	PFS	0.71 [0.31, 1.11]
Kingston et al,[19] 2021	*PIK3CA* (multiple)	All	PFS	3.15 [-2.07, 8.37]
Kumar et al,[20] 2018	*ESR1, PIK3CA, or TP53*	HR+	PFS	3.80 [0.95, 6.65]
Lee et al,[21] 2023	*PIK3CA*	HER2+	OS	2.86 [0.13, 5.59]
Lee et al,[21] 2023	*PIK3CA*	HER2+	PFS	2.07 [0.82, 3.32]
Lee et al,[21] 2023	*TP53*	HER2+	OS	2.61 [0.38, 4.84]
Lee et al,[21] 2023	*TP53*	HER2+	PFS	2.17 [1.11, 3.23]
Liu et al,[23] 2021	*ESR1*	HR+, HER2-	PFS	1.20 [-1.58, 3.98]
Liu et al,[23] 2021	*PTEN*	HR+, HER2-	PFS	1.28 [-3.77, 6.33]
Liu et al,[23] 2021	*ESR1*	HR+, HER2-	PFS	2.40 [0.15, 4.65]
Liu et al,[23] 2021	*PTEN*	HR+, HER2-	PFS	3.50 [-1.89, 8.89]
Page et al,[28] 2017	*ESR1*	All	OS	25.61 [-43.69, 94.91]
Page et al,[29] 2021	*ESR1/PIK3CA/TP53*	All	OS	3.46 [1.19, 5.73]
Pascual et al,[30] 2023	*TP53*	HR+, HER2-	OS	2.89 [1.36, 4.42]
Pascual et al,[30] 2023	*TP53*	HR+, HER2-	PFS	1.84 [1.17, 2.51]
Raimondi et al,[31] 2021	*KRAS*	HR+, HER2-	PFS	20.75 [3.07, 38.43]
Schiavon et al,[32] 2015	*ESR1*	All	PFS	3.10 [-7.50, 13.70]
Sharma et al,[33] 2021	*PIK3CA*	All	PFS	0.44 [0.08, 0.80]
Tang et al,[35] 2022	*PIK3CA*	HR+, HER2-	PFS	2.04 [0.77, 3.31]
Tang et al,[35] 2022	*FGFR*	HR+, HER2-	PFS	1.90 [0.62, 3.18]
Tang et al,[35] 2022	*ESR1 + GATA3*	HR+, HER2-	PFS	1.87 [0.91, 2.83]
Tsiji et al,[36] 2022	*ESR1*	HR+, HER2-	PFS	1.00 [0.00, 2.00]
Tsiji et al,[36] 2022	*PIK3CA*	HR+, HER2-	PFS	0.20 [-0.07, 0.47]
Wang et al,[37] 2023	*FGFR*	HR+, HER2-	DFS	2.33 [0.55, 4.11]
Yi et al,[39] 2020	*ERBB2*	All	PFS	3.37 [-0.09, 6.83]
Yi et al,[40] 2020	*PIK3CA*	All	DFS	0.18 [-0.27, 0.63]
**Total**				**1.48 [1.15, 1.80]**

Heterogeneity: $\tau^2 = 0.43$, $I^2 = 71.08\%$, $H^2 = 3.46$
Test of overall effect: $z = 8.98$, $p = <0.001$

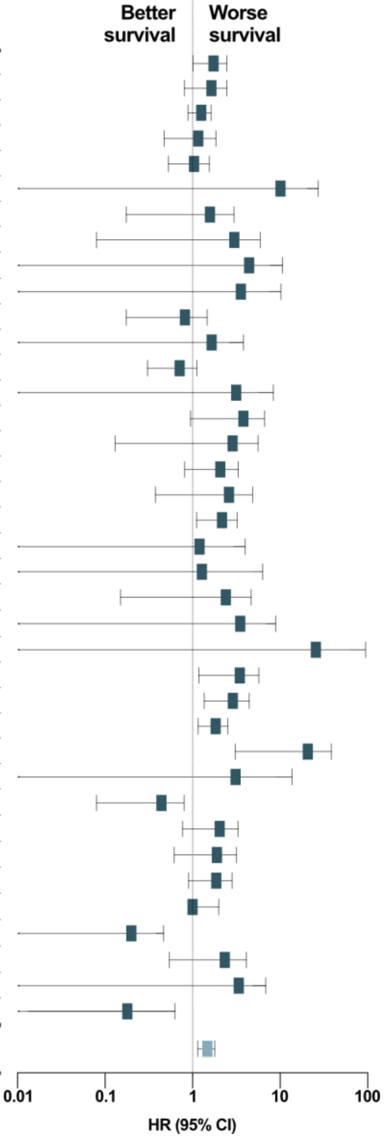

© 2024 Dickinson K et al. *JAMA Network Open*.

B. Retrospective

Source	Mutation	Breast cancer subtype	Outcome tested	HR [95% CI]
Bai et al,[5] 2021	*TP53*	All	OS	1.99 [0.97, 3.01]
Bai et al,[5] 2021	*TP53*	All	DFS	1.89 [1.19, 2.59]
Bai et al,[5] 2021	*TP53*	HER2+	OS	2.47 [-0.49, 5.43]
Bai et al,[5] 2021	*TP53*	HER2+	DFS	1.10 [0.04, 2.16]
Bai et al,[5] 2021	*TP53*	HR+, HER2-	OS	1.20 [0.28, 2.12]
Bai et al,[5] 2021	*TP53*	HR+, HER2-	DFS	1.57 [0.79, 2.35]
Bai et al,[5] 2021	*TP53*	TNBC	OS	2.42 [-0.83, 5.67]
Bai et al,[5] 2021	*TP53*	TNBC	DFS	2.17 [0.20, 4.14]
Chandarlapaty et al,[6] 2016	*ESR1*	HR+, HER2-	OS	1.62 [1.25, 1.99]
Chin et al,[8] 2022	*ESR1*	HR+, HER2-	PFS	5.25 [-3.25, 13.54]
Chin et al,[8] 2022	*PIK3CA*	HR+, HER2-	PFS	1.16 [-0.27, 2.59]
Clatot et al,[9] 2016	*ESR1*	All	OS	1.90 [0.95, 2.85]
Clatot et al,[9] 2016	*ESR1*	All	PFS	1.70 [1.00, 2.40]
Crucitta et al,[11] 2023	*ESR1*	HR+	DFS	3.18 [0.03, 6.33]
Del Re et al,[12] 2020	*PIK3CA*	All	PFS	2.93 [0.07, 5.79]
Fribbens et al,[13] 2016 (SoFEA)	*ESR1*	HR+, HER2-	OS	1.65 [0.37, 2.93]
Fribbens et al,[13] 2016 (PALOMA3)	*ESR1*	HR+, HER2-	OS	1.46 [0.98, 1.94]
Fribbens et al,[13] 2016 (SoFEA)	*ESR1*	HR+, HER2-	PFS	2.12 [0.81, 3.43]
Gyanchandani et al,[17] 2017	*ESR1*	HR+, HER2-	OS	0.32 [-0.72, 1.36]
Gyanchandani et al,[17] 2017	*ESR1*	HR+, HER2-	PFS	0.25 [-0.19, 0.69]
Li et al,[22] 2020	*TP53*	All	OS	4.31 [-1.45, 10.07]
Liu et al,[24] 2022	*TP53*	All	PFS	1.00 [0.23, 1.77]
Liu et al,[24] 2022	*TP53*	All	PFS	1.19 [0.31, 2.07]
Moynahan et al,[26] 2017	*PIK3CA*	HR+, HER2-	OS	1.32 [0.98, 1.66]
Moynahan et al,[26] 2017	*PIK3CA*	HR+, HER2-	PFS	1.26 [0.98, 1.54]
O'Leary et al,[27] 2018	*ESR1*	HR+, HER2-	PFS	1.58 [0.88, 2.28]
Spoerke et al,[34] 2016	*ESR1* (1 mutation)	HR+, HER2-	PFS	0.89 [0.29, 1.49]
Spoerke et al,[34] 2016	*ESR1* (>1 mutation)	HR+, HER2-	PFS	1.39 [0.29, 2.49]
Yi et al,[38] 2020	*PIK3CA*	All	PFS	1.20 [-0.31, 2.71]
Yi et al,[38] 2020	*TP53*	All	PFS	1.32 [1.06, 1.58]
Zhang et al,[41] 2022	*TP53*	All	DFS	3.14 [0.23, 6.05]
Zhang et al,[41] 2022	*CTCF/GNAS*	All	DFS	7.23 [-6.57, 21.03]
Zhang et al,[41] 2022	*TOP1*	All	DFS	7.21 [-6.94, 21.36]
Zhang et al,[41] 2022	*NOTCH2*	All	DFS	3.44 [-2.13, 9.01]
**Total**				**1.37 [1.17, 1.56]**

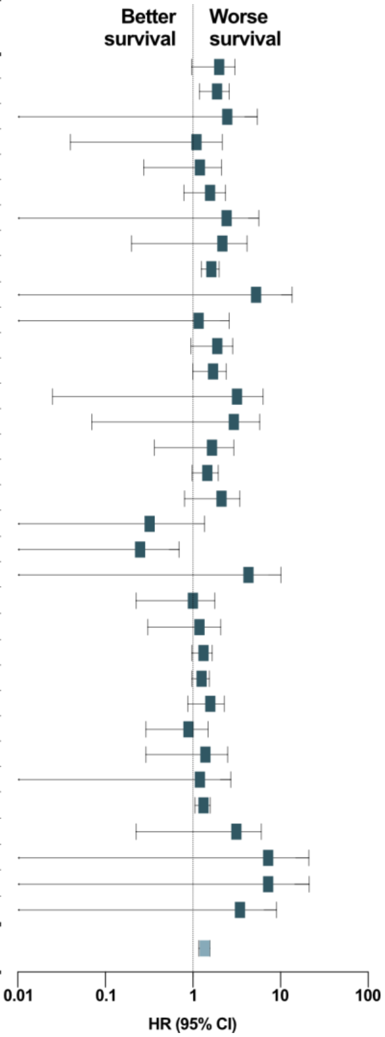

Heterogeneity: $\tau^2 = 0.10$, $I^2 = 43.72\%$, $H^2 = 1.78$
Test of overall effect: z = 13.68, p = <0.001

© 2024 Dickinson K et al. *JAMA Network Open*.

eFigure 5. Subgroup Analysis of ctDNA Detection Methods

A. NGS

Source	Mutation	Breast cancer subtype	Outcome tested	HR [95% CI]
Bai et al,[5] 2021	*TP53*	All	OS	1.99 [0.97, 3.01]
Bai et al,[5] 2021	*TP53*	All	DFS	1.89 [1.19, 2.59]
Bai et al,[5] 2021	*TP53*	HER2+	OS	2.47 [-0.49, 5.43]
Bai et al,[5] 2021	*TP53*	HER2+	DFS	1.10 [0.04, 2.16]
Bai et al,[5] 2021	*TP53*	HR+, HER2-	OS	1.20 [0.28, 2.12]
Bai et al,[5] 2021	*TP53*	HR+, HER2-	DFS	1.57 [0.79, 2.35]
Bai et al,[5] 2021	*TP53*	TNBC	OS	2.42 [-0.83, 5.67]
Bai et al,[5] 2021	*TP53*	TNBC	DFS	2.17 [0.20, 4.14]
Chen et al,[7] 2023	*PIK3CA*	HR+, HER2-	PFS	1.74 [1.01, 2.47]
Chen et al,[7] 2023	*TP53*	HR+, HER2-	PFS	1.64 [0.81, 2.47]
Chin et al,[8] 2022	*ESR1*	HR+, HER2-	PFS	5.25 [-3.25, 13.54]
Chin et al,[8] 2022	*PIK3CA*	HR+, HER2-	PFS	1.16 [-0.27, 2.59]
Guan et al,[16] 2023	*ERBB2*	All	OS	10.10 [-7.04, 27.24]
Guan et al,[16] 2023	*PIK3CA*	All	OS	1.58 [0.18, 2.98]
Guan et al,[16] 2023	*PIK3CA*	All	PFS	2.99 [0.08, 5.90]
Guan et al,[16] 2023	*ERBB2*	All	PFS	4.43 [-1.80, 10.66]
Hu et al,[18] 2021	*TP53*	All	PFS	3.57 [-3.09, 10.23]
Kingston et al,[19] 2021	*ESR1*	All	OS	0.82 [0.18, 1.46]
Kingston et al,[19] 2021	*MAPK*	All	OS	1.65 [-0.51, 3.81]
Kingston et al,[19] 2021	*PIK3CA* (single)	All	PFS	0.71 [0.31, 1.11]
Kingston et al,[19] 2021	*PIK3CA* (multiple)	All	PFS	3.15 [-2.07, 8.37]
Lee et al,[21] 2023	*PIK3CA*	HER2+	OS	2.86 [0.13, 5.59]
Lee et al,[21] 2023	*PIK3CA*	HER2+	PFS	2.07 [0.82, 3.32]
Lee et al,[21] 2023	*TP53*	HER2+	OS	2.61 [0.38, 4.84]
Lee et al,[21] 2023	*TP53*	HER2+	PFS	2.17 [1.11, 3.23]
Li et al,[22] 2020	*TP53*	All	OS	4.31 [-1.45, 10.07]
Liu et al,[23] 2021	*ESR1*	HR+, HER2-	PFS	1.20 [-1.58, 3.98]
Liu et al,[24] 2022	*TP53*	All	PFS	1.00 [0.23, 1.77]
Liu et al,[23] 2021	*PTEN*	HR+, HER2-	PFS	1.28 [-3.77, 6.33]
Liu et al,[24] 2022	*TP53*	All	PFS	1.19 [0.31, 2.07]
Liu et al,[23] 2021	*ESR1*	HR+, HER2-	PFS	2.40 [0.15, 4.65]
Liu et al,[23] 2021	*PTEN*	HR+, HER2-	PFS	3.50 [-1.89, 8.89]
Page et al,[29] 2021	*ESR1/PIK3CA/TP53*	All	OS	3.46 [1.19, 5.73]
Pascual et al,[30] 2023	*TP53*	HR+, HER2-	OS	2.89 [1.36, 4.42]
Pascual et al,[30] 2023	*TP53*	HR+, HER2-	PFS	1.84 [1.17, 2.51]
Sharma et al,[33] 2021	*PIK3CA*	All	PFS	0.44 [0.08, 0.80]
Tang et al,[35] 2022	*PIK3CA*	HR+, HER2-	PFS	2.04 [0.77, 3.31]
Tang et al,[35] 2022	*FGFR*	HR+, HER2-	PFS	1.90 [0.62, 3.18]
Tang et al,[35] 2022	*ESR1* + *GATA3*	HR+, HER2-	PFS	1.87 [0.91, 2.83]
Tsiji et al,[36] 2022	*ESR1*	HR+, HER2-	PFS	1.00 [0.00, 2.00]
Tsiji et al,[36] 2022	*PIK3CA*	HR+, HER2-	PFS	0.20 [-0.07, 0.47]
Wang et al,[37] 2023	*FGFR*	HR+, HER2-	DFS	2.33 [0.55, 4.11]
Yi et al,[38] 2020	*PIK3CA*	All	PFS	1.20 [-0.31, 2.71]
Yi et al,[38] 2020	*TP53*	All	PFS	1.32 [1.06, 1.58]
Yi et al,[39] 2020	*ERBB2*	All	PFS	3.37 [-0.09, 6.83]
Yi et al,[40] 2020	*PIK3CA*	All	DFS	0.18 [-0.27, 0.63]
Zhang et al,[41] 2022	*TP53*	All	DFS	3.14 [0.23, 6.05]
Zhang et al,[41] 2022	*CTCF/GNAS*	All	DFS	7.23 [-6.57, 21.03]
Zhang et al,[41] 2022	*TOP1*	All	DFS	7.21 [-6.94, 21.36]
Zhang et al,[41] 2022	*NOTCH2*	All	DFS	3.44 [-2.13, 9.01]
**Total**				**1.48 [1.22, 1.74]**

Heterogeneity: $\tau^2 = 0.34$, $I^2 = 63.56\%$, $H^2 = 2.74$

Test of overall effect: $z = 11.08$, $p = <0.001$

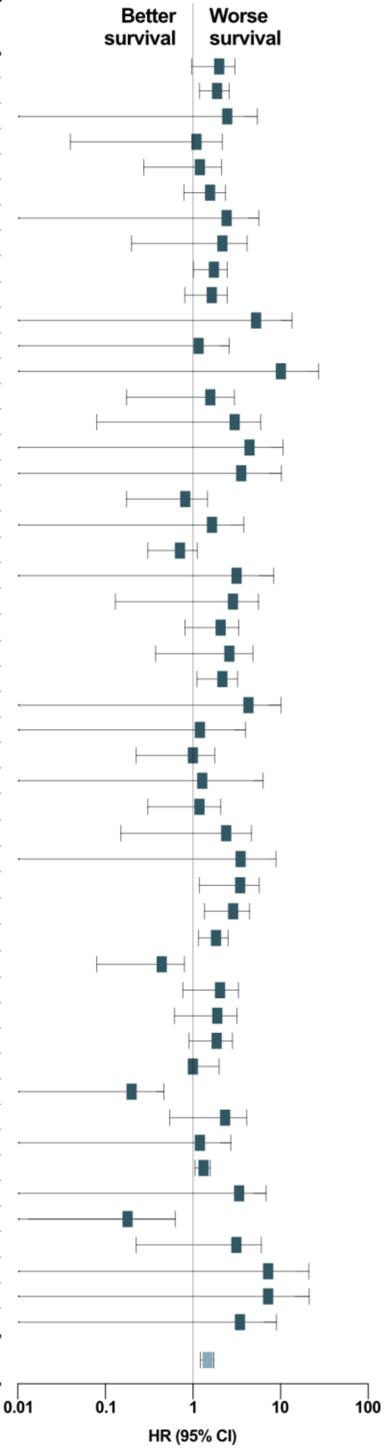

© 2024 Dickinson K et al. *JAMA Network Open*.

B. dPCR

Source	Mutation	Breast cancer subtype	Outcome tested	HR [95% CI]
Chandarlapaty et al,[6] 2016	*ESR1*	HR+, HER2-	OS	1.62 [1.25, 1.99]
Clatot et al,[9] 2016	*ESR1*	All	OS	1.90 [0.95, 2.85]
Clatot et al,[9] 2016	*ESR1*	All	PFS	1.70 [1.00, 2.40]
Cristofanilli et al,[10] 2016	*PIK3CA*	HR+, HER2-	PFS	1.26 [0.89, 1.63]
Crucitta et al,[11] 2023	*ESR1*	HR+	DFS	3.18 [0.03, 6.33]
Del Re et al,[12] 2020	*PIK3CA*	All	PFS	2.93 [0.07, 5.79]
Fribbens et al,[13] 2016 (SoFEA)	*ESR1*	HR+, HER2-	OS	1.65 [0.37, 2.93]
Fribbens et al,[14] 2018	*KRAS*	All	OS	1.16 [0.48, 1.84]
Fribbens et al,[14] 2018	*KRAS*	All	PFS	1.04 [0.53, 1.55]
Fribbens et al,[13] 2016 (PALOMA3)	*ESR1*	HR+, HER2-	OS	1.46 [0.98, 1.94]
Fribbens et al,[13] 2016 (SoFEA)	*ESR1*	HR+, HER2-	PFS	2.12 [0.81, 3.43]
Gyanchandani et al,[17] 2017	*ESR1*	HR+, HER2-	OS	0.32 [-0.72, 1.36]
Gyanchandani et al,[17] 2017	*ESR1*	HR+, HER2-	PFS	0.25 [-0.19, 0.69]
Moynahan et al,[26] 2017	*PIK3CA*	HR+, HER2-	OS	1.32 [0.98, 1.66]
Moynahan et al,[26] 2017	*PIK3CA*	HR+, HER2-	PFS	1.26 [0.98, 1.54]
O'Leary et al,[27] 2018	*ESR1*	HR+, HER2-	PFS	1.58 [0.88, 2.28]
Page et al,[28] 2017	*ESR1*	All	OS	25.61 [-43.69, 94.91]
Raimondi et al,[31] 2021	*KRAS*	HR+, HER2-	PFS	20.75 [3.07, 38.43]
Schiavon et al,[32] 2015	*ESR1*	All	PFS	3.10 [-7.50, 13.70]
Spoerke et al,[34] 2016	*ESR1* (1 mutation)	HR+, HER2-	PFS	0.89 [0.29, 1.49]
Spoerke et al,[34] 2016	*ESR1* (>1 mutation)	HR+, HER2-	PFS	1.39 [0.29, 2.49]
**Total**				**1.28 [1.05, 1.50]**

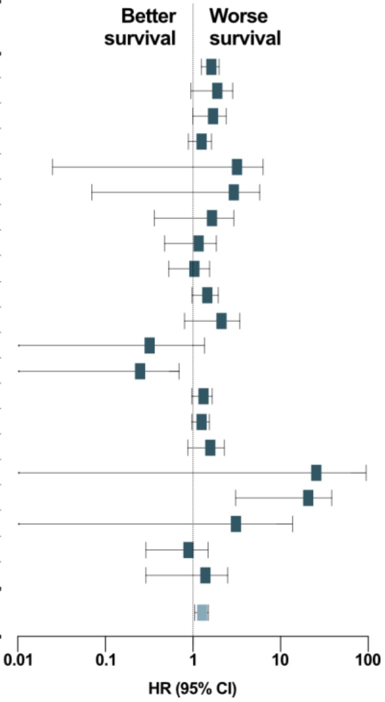

Heterogeneity: $\tau^2 = 0.11$, $I^2 = 53.92\%$, $H^2 = 2.17$
Test of overall effect: $z = 11.13$, $p = <0.001$

© 2024 Dickinson K et al. *JAMA Network Open*.

eFigure 6. Subgroup Analysis of Blood Collection Tube Used

A. EDTA

Source	Mutation	Breast cancer subtype	Outcome tested	HR [95% CI]
Bai et al,[5] 2021	*TP53*	All	OS	1.99 [0.97, 3.01]
Bai et al,[5] 2021	*TP53*	All	DFS	1.89 [1.19, 2.59]
Bai et al,[5] 2021	*TP53*	HER2+	OS	2.47 [-0.49, 5.43]
Bai et al,[5] 2021	*TP53*	HER2+	DFS	1.10 [0.04, 2.16]
Bai et al,[5] 2021	*TP53*	HR+, HER2-	OS	1.20 [0.28, 2.12]
Bai et al,[5] 2021	*TP53*	HR+, HER2-	DFS	1.57 [0.79, 2.35]
Bai et al,[5] 2021	*TP53*	TNBC	OS	2.42 [-0.83, 5.67]
Bai et al,[5] 2021	*TP53*	TNBC	DFS	2.17 [0.20, 4.14]
Chandarlapaty et al,[6] 2016	*ESR1*	HR+, HER2-	OS	1.62 [1.25, 1.99]
Chin et al,[8] 2022	*ESR1*	HR+, HER2-	PFS	5.25 [-3.25, 13.54]
Chin et al,[8] 2022	*PIK3CA*	HR+, HER2-	PFS	1.16 [-0.27, 2.59]
Crucitta et al,[11] 2023	*ESR1*	HR+	DFS	3.18 [0.03, 6.33]
Del Re et al,[12] 2020	*PIK3CA*	All	PFS	2.93 [0.07, 5.79]
Fribbens et al,[13] 2016 (SoFEA)	*ESR1*	HR+, HER2-	OS	1.65 [0.37, 2.93]
Fribbens et al,[14] 2018	*KRAS*	All	OS	1.16 [0.48, 1.84]
Fribbens et al,[14] 2018	*KRAS*	All	PFS	1.04 [0.53, 1.55]
Fribbens et al,[13] 2016 (PALOMA3)	*ESR1*	HR+, HER2-	OS	1.46 [0.98, 1.94]
Fribbens et al,[13] 2016 (SoFEA)	*ESR1*	HR+, HER2-	PFS	2.12 [0.81, 3.43]
Fuentes-Antrás et al,[15] 2023	*PIK3CA*	HR+, HER2-	PFS	2.14 [0.92, 3.36]
Fuentes-Antrás et al,[15] 2023	*TP53*	HR+, HER2-	PFS	2.40 [-0.17, 4.97]
Li et al,[22] 2020	*TP53*	All	OS	4.31 [-1.45, 10.07]
Mosele et al,[25] 2020	*PIK3CA*	HR+, HER2-	OS	1.44 [0.94, 1.94]
Moynahan et al,[26] 2017	*PIK3CA*	HR+, HER2-	OS	1.32 [0.98, 1.66]
Moynahan et al,[26] 2017	*PIK3CA*	HR+, HER2-	PFS	1.26 [0.98, 1.54]
O'Leary et al,[27] 2018	*ESR1*	HR+, HER2-	PFS	1.58 [0.88, 2.28]
Page et al,[28] 2017	*ESR1*	All	OS	25.61 [-43.69, 94.91]
Page et al,[29] 2021	*ESR1/PIK3CA/TP53*	All	OS	3.46 [1.19, 5.73]
Schiavon et al,[32] 2015	*ESR1*	All	PFS	3.10 [-7.50, 13.70]
Spoerke et al,[34] 2016	*ESR1* (1 mutation)	HR+, HER2-	PFS	0.89 [0.29, 1.49]
Spoerke et al,[34] 2016	*ESR1* (>1 mutation)	HR+, HER2-	PFS	1.39 [0.29, 2.49]
Tsiji et al,[36] 2022	*ESR1*	HR+, HER2-	PFS	1.00 [0.00, 2.00]
Tsiji et al,[36] 2022	*PIK3CA*	HR+, HER2-	PFS	0.20 [-0.07, 0.47]
Zhang et al,[41] 2022	*TP53*	All	DFS	3.14 [0.23, 6.05]
Zhang et al,[41] 2022	*CTCF/GNAS*	All	DFS	7.23 [-6.57, 21.03]
Zhang et al,[41] 2022	*TOP1*	All	DFS	7.21 [-6.94, 21.36]
Zhang et al,[41] 2022	*NOTCH2*	All	DFS	3.44 [-2.13, 9.01]
**Total**				**1.40 [1.18, 1.63]**

Heterogeneity: $\tau^2 = 0.16$, $I^2 = 52.85\%$, $H^2 = 2.12$
Test of overall effect: $z = 12.23$, $p = <0.001$

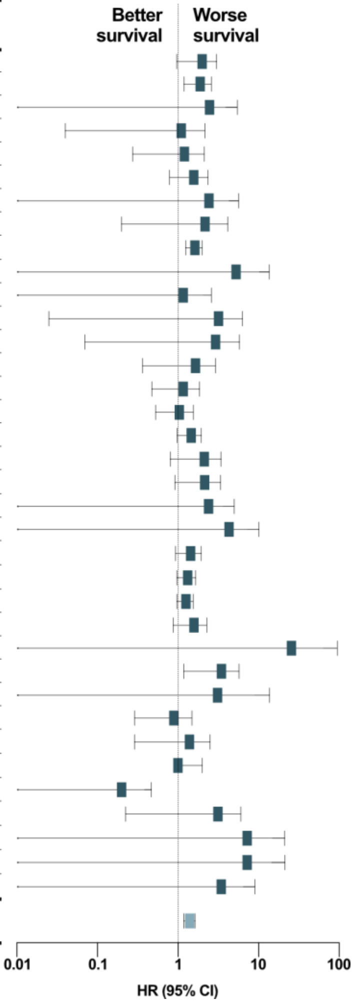

© 2024 Dickinson K et al. *JAMA Network Open*.

B. Streck

Source	Mutation	Breast cancer subtype	Outcome tested	HR [95% CI]
Chen et al,[7] 2023	*PIK3CA*	HR+, HER2-	PFS	1.74 [1.01, 2.47]
Chen et al,[7] 2023	*TP53*	HR+, HER2-	PFS	1.64 [0.81, 2.47]
Gyanchandani et al,[17] 2017	*ESR1*	HR+, HER2-	OS	0.32 [-0.72, 1.36]
Gyanchandani et al,[17] 2017	*ESR1*	HR+, HER2-	PFS	0.25 [-0.19, 0.69]
Kingston et al,[19] 2021	*ESR1*	All	OS	0.82 [0.18, 1.46]
Kingston et al,[19] 2021	*MAPK*	All	OS	1.65 [-0.51, 3.81]
Kingston et al,[19] 2021	*PIK3CA* (single)	All	PFS	0.71 [0.31, 1.11]
Kingston et al,[19] 2021	*PIK3CA* (multiple)	All	PFS	3.15 [-2.07, 8.37]
Kumar et al,[20] 2018	*ESR1, PIK3CA, or TP53*	HR+	PFS	3.80 [0.95, 6.65]
Lee et al,[21] 2023	*PIK3CA*	HER2+	OS	2.86 [0.13, 5.59]
Lee et al,[21] 2023	*PIK3CA*	HER2+	PFS	2.07 [0.82, 3.32]
Lee et al,[21] 2023	*TP53*	HER2+	OS	2.61 [0.38, 4.84]
Lee et al,[21] 2023	*TP53*	HER2+	PFS	2.17 [1.11, 3.23]
Liu et al,[24] 2022	*TP53*	All	PFS	1.00 [0.23, 1.77]
Liu et al,[24] 2022	*TP53*	All	PFS	1.19 [0.31, 2.07]
Pascual et al,[30] 2023	*TP53*	HR+, HER2-	OS	2.89 [1.36, 4.42]
Pascual et al,[30] 2023	*TP53*	HR+, HER2-	PFS	1.84 [1.17, 2.51]
Tang et al,[35] 2022	*PIK3CA*	HR+, HER2-	PFS	2.04 [0.77, 3.31]
Tang et al,[35] 2022	*FGFR*	HR+, HER2-	PFS	1.90 [0.62, 3.18]
Tang et al,[35] 2022	*ESR1 + GATA3*	HR+, HER2-	PFS	1.87 [0.91, 2.83]
Wang et al,[37] 2023	*FGFR*	HR+, HER2-	DFS	2.33 [0.55, 4.11]
Yi et al,[38] 2020	*PIK3CA*	All	PFS	1.20 [-0.31, 2.71]
Yi et al,[38] 2020	*TP53*	All	PFS	1.32 [1.06, 1.58]
Yi et al,[39] 2020	*ERBB2*	All	PFS	3.37 [-0.09, 6.83]
Yi et al,[40] 2020	*PIK3CA*	All	DFS	0.18 [-0.27, 0.63]
**Total**				**1.41 [1.07, 1.74]**

Heterogeneity: $\tau^2 = 0.38$, $I^2 = 71.67\%$, $H^2 = 3.53$
Test of overall effect: z = 8.27, p = <0.001

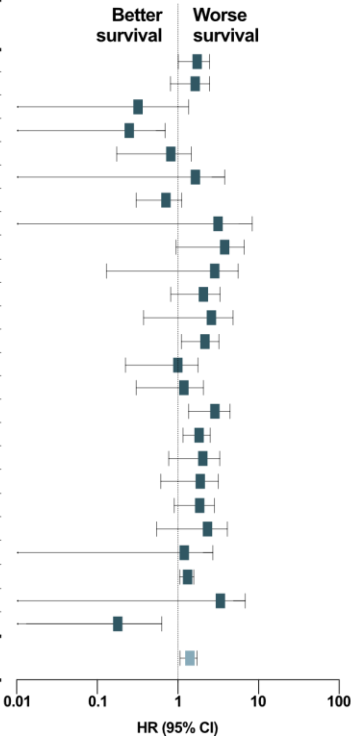

C. Other

Source	Mutation	Breast cancer subtype	Outcome tested	HR [95% CI]
Clatot et al,[9] 2016	*ESR1*	All	OS	1.90 [0.95, 2.85]
Clatot et al,[9] 2016	*ESR1*	All	PFS	1.70 [1.00, 2.40]
Cristofanilli et al,[10] 2016	*PIK3CA*	HR+, HER2-	PFS	1.26 [0.89, 1.63]
Guan et al,[16] 2023	*ERBB2*	All	OS	10.10 [-7.04, 27.24]
Guan et al,[16] 2023	*PIK3CA*	All	OS	1.58 [0.18, 2.98]
Guan et al,[16] 2023	*PIK3CA*	All	PFS	2.99 [0.08, 5.90]
Guan et al,[16] 2023	*ERBB2*	All	PFS	4.43 [-1.80, 10.66]
Hu et al,[18] 2021	*TP53*	All	PFS	3.57 [-3.09, 10.23]
Liu et al,[23] 2021	*ESR1*	HR+, HER2-	PFS	1.20 [-1.58, 3.98]
Liu et al,[23] 2021	*PTEN*	HR+, HER2-	PFS	1.28 [-3.77, 6.33]
Liu et al,[23] 2021	*ESR1*	HR+, HER2-	PFS	2.40 [0.15, 4.65]
Liu et al,[23] 2021	*PTEN*	HR+, HER2-	PFS	3.50 [-1.89, 8.89]
Raimondi et al,[31] 2021	*KRAS*	HR+, HER2-	PFS	20.75 [3.07, 38.43]
Sharma et al,[33] 2021	*PIK3CA*	All	PFS	0.44 [0.08, 0.80]
**Total**				**1.47 [0.92, 2.02]**

Heterogeneity: $\tau^2 = 0.32$, $I^2 = 55.14\%$, $H^2 = 2.23$
Test of overall effect: z = 5.27, p = <0.001

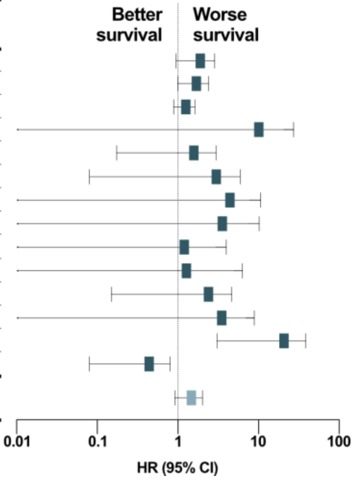

© 2024 Dickinson K et al. *JAMA Network Open*.

eFigure 7. Funnel Plot to Assess Publication Bias. Kendall's score = 180.00, SE = 218.611; z = 0.82; Prob > |z| = 0.4129.

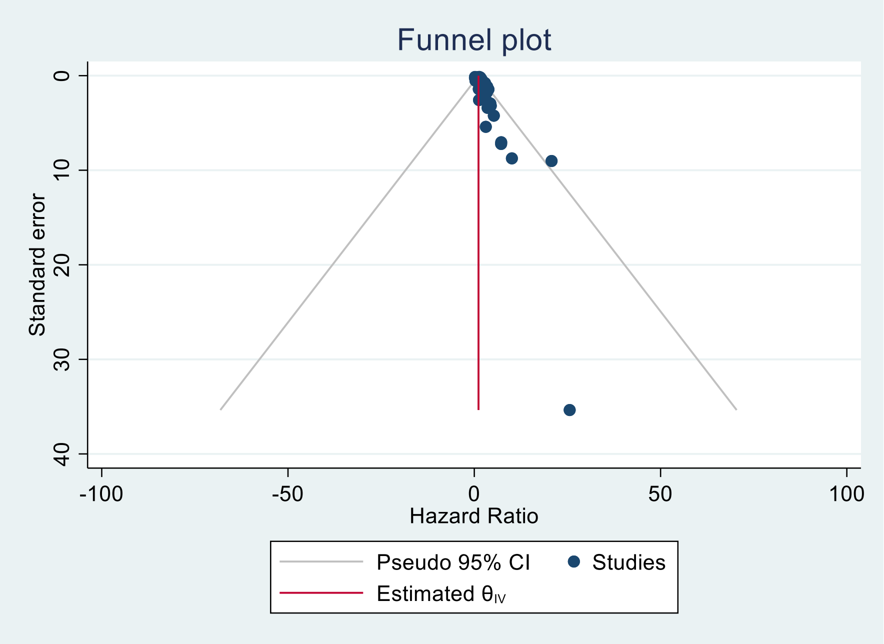

© 2024 Dickinson K et al. *JAMA Network Open*.
