# Supplementary material for: Circulating Tumor DNA and Survival in Metastatic Breast Cancer: A Systematic Review and Meta-Analysis
Source: JAMA Netw Open. 2024 Sep 5;7(9):e2431722. doi: 10.1001/jamanetworkopen.2024.31722 (PMC11378006; doi:10.1001/jamanetworkopen.2024.31722)
Supplement: Supplement 3. — Data Sharing Statement [file jamanetwopen-e2431722-s003.pdf]

Data Sharing Statement

Dickinson. Circulating Tumor DNA and Survival in Metastatic Breast Cancer. *JAMA Netw Open*. Published September 05, 2024. doi:10.1001/jamanetworkopen.2024.31722

Data
Data available: Yes
Data types: Other (please specify)
Additional Information: As this is a meta-analysis performed on data obtained from published studies, we do not have access to individual participant data. However, upon request, we can provide the data table used to generate our results.
How to access data: Request for data must be sent to the corresponding author, Dr. Kyle Dickinson (kyle.dickinson@mail.mcgill.ca).
When available: With publication

Supporting Documents
Document types: None

Additional Information
Who can access the data: We do not have any supporting documents to make available.
Types of analyses: Not applicable.
Mechanisms of data availability: Not applicable.
